# Nano-in-Micro GelMA depots assist electro-thermal-immuno orchestral treatment for solid triple negative breast tumor

**DOI:** 10.1016/j.mtbio.2026.102848

**Published:** 2026-01-30

**Authors:** Jiachen Li, Yaping Zhuang, Huijie Han, Yuewen Zhu, Chao Lin, Rui Wang, Ana Catarina Rodrigues da Silva, Marc C.A. Stuart, Guimei Jiang, Siyu Fan, Romana Schirhagl, Mohammad-Ali Shahbazi, Lígia Raquel Marona Rodrigues, Wenguo Cui, Hélder A. Santos

**Affiliations:** a Department of Orthopaedics, Shanghai Key Laboratory for Prevention and Treatment of Bone and Joint Diseases, Shanghai Institute of Traumatology and Orthopaedics, Ruijin Hospital, Shanghai Jiao Tong University School of Medicine, 197 Ruijin 2nd Road, Shanghai, 200025, China; b Department of Biomaterials and Biomedical Technology, The Personalized Medicine Research Institute (PRECISION), University Medical Center Groningen, University of Groningen, Groningen, 9713 AV, the Netherlands; c College of Chemistry and Life Science, Beijing University of Technology, 100 Pingleyuan, Chaoyang District, Beijing, 100124, China; d Wuxi School of Medicine, Jiangnan University, Wuxi, Jiangsu, 214000, China; e The International Peace Maternity and Child Health Hospital, School of Medicine, Shanghai Jiao Tong University, Shanghai, 200030, China; f Groningen Biomolecular Sciences and Biotechnology Institute, Faculty of Science and Engineering, University of Groningen, Nijenborgh 7, Groningen, 9747 AG, the Netherlands; g CEB-Centre of Biological Engineering, Universidade do Minho, Campus de Gualtar, Braga, 4710-057, Portugal; h LABBELS, Associate Laboratory, Braga, Guimarães, Portugal

**Keywords:** TNBC, Nano-in-Micro, Microsphere depot, Local treatments, Combination therapy

## Abstract

Triple-negative breast cancer (TNBC) exhibits high local-recurrence risk despite modern systemic therapy assisted with surgery or irradiation therapy. Here, we report an injectable nano-in-micro microsphere depot (cPAG) that integrates conductivity enhancement, photothermal conversion, O_2_ and reactive oxygen species (ROS) generation, and innate immune agonist co-delivery to support staged, local multimodal therapy. Monodispersed Au@GelMA microspheres prepared *via* microfluidics and photocrosslinking reduced high electrostatic resistance of GelMA hydrogels and provided stable 808 nm laser-responsive heating. The porous surface possessed abundant electrostatic adsorption sites for loading MnO_x_ nanoflowers and anionic stimulator of interferon genes (STING) agonist. MnO_x_ nanoflowers catalyzed H_2_O_2_ to generate O_2,_ produced free radical signals and increased pro-inflammatory cytokines secretion *in vitro*. Co-delivery of agonist and MnO_x_ nanoparticles further increased interferon-β secretion, consistent with induction of type I interferon response. In a 4T1 residual-tumor model established by partial tumor resection, a staged regimen consisting of cPAG-assisted irreversible electroporation followed by cPAG-mediated photothermal therapy showed the strongest suppression of local tumor regrowth among tested groups, with maintained body weight during the study window. Overall, cPAG provides a modular nano-in-micro depot strategy to integrate multiple local treatments for postoperative control of TNBC tumor.

## Highlights

• A nano-in-micro c-di-AMP@PM@Au@GelMA microsphere depot was developed for triple negative breast cancer local comprehensive intervention.
• Depots integrate electrical conductivity tuning, photothermal effect, oxygen generation, ROS amplification, and immune activation.
• Microsphere depot-based multimodal local regimen inhibited tumor regrowth and prolonged survival while stabilizing body weight *in vivo*.

## Graphical abstract

**Figure:**
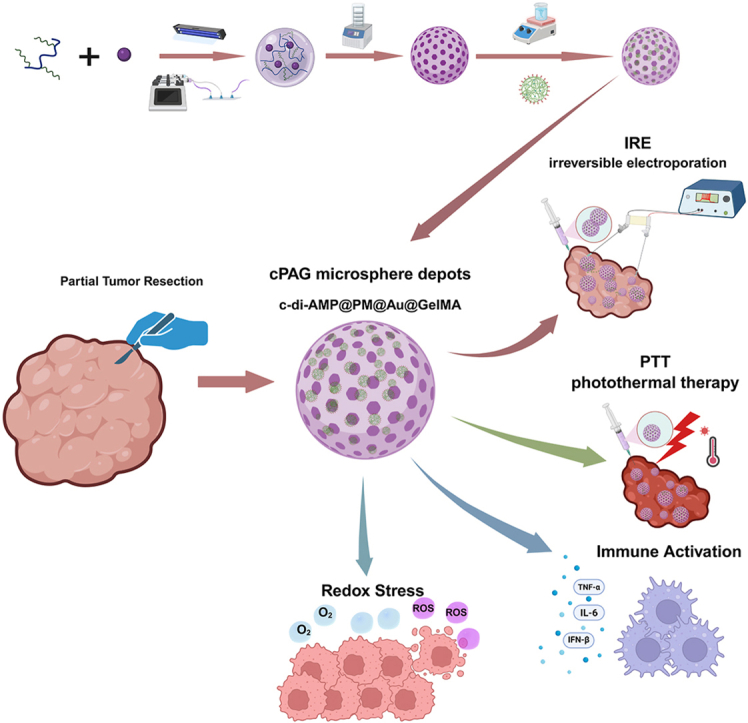

## 1 Introduction

In 2022, there were approximately 2.3 million new cases of breast cancer and more than 666,000 deaths [1], making it the most diagnosed cancer in women and leading cause of female cancer death. Among these, triple-negative breast cancer (TNBC) lacking estrogen receptor (ER), progesterone receptor (PR) and human epidermal growth factor receptor 2 (HER2) targets shows aggressive biology with early distant relapses and high post-treatment recurrence where ∼30–50 % of operable patients recur within 5 years [[2], [3], [4]]. Although modern systemic therapy (*e.g.*, neoadjuvant chemotherapy plus pembrolizumab) has improved pathologic complete response (pCR) and overall survival [5], TNBC still carries a worse prognosis and higher mortality than other breast cancer subtypes, underscoring it as one of the most dangerous forms [3]. Moreover, systemic therapy as dominated treatment method still faces several clinical challenges [6], *e.g.*, the systemic side effects like myelosuppression or neuropathy, or the possible residual tumor nests at cut surface (non-pCR cases) driving recurrences.

For operable TNBC cases, systemic therapy integrates with local therapy like surgery or radiation therapy [7,8]. To be specific, surgery can remove macroscopic disease and allow real-time tumor margin management [9]. Also, non-thermal irreversible electroporation (IRE) therapy can ablate tumor while preserving skin, glandular lobules and stromal architecture, preserving collagen-rich structure in delicate breast planes for improved organ-sparing cosmesis [[10], [11], [12]]. Adjunct local ablation like repeatable and focal photothermal therapy (PTT) can further cauterize the resection cavity, enabling better margin mopping up and reducing local regrowth after surgery [13,14]. Furthermore, IRE therapy and PTT both can trigger specific anti-tumor immune response, activating downstream cytotoxic T cells to inhibit tumor recurrence and metastasis [15,16]. Overall, combining local therapies for TNBC provide immediate cytoreduction and margin control at the tumor bed, better cosmetic and functional preservation (*e.g.*, organ form and cosmesis), lower systemic toxicity, extending disease control without forcing systemic regimen changes since systemic therapy remains essential for survival [14,17,18]. Hence, to investigate the therapeutic effect of local treatment on TNBC solid tumor, surgical therapy combining IRE therapy and then assisted by PTT was selected as a trial therapeutic modality for this study.

However, IRE therapy is constrained by the high electrical impedance of soft tissue [19,20]. Near-infrared (NIR) laser-based PTT only provides transient hyperthermal [21,22], and hypoxia in tumor tissue blunts the efficacy of IRE [23,24], PTT [25,26]. Besides, hypoxia drives an immunosuppressive tumor microenvironment (TME) [27,28]. Consequently, these challenges motivate local delivery strategies that can modulate electrical properties to support IRE, provide controllable photothermal conversion for PTT, and introduce catalytic and immunostimulatory functions in a locally retained format.

**Fig. 1:**
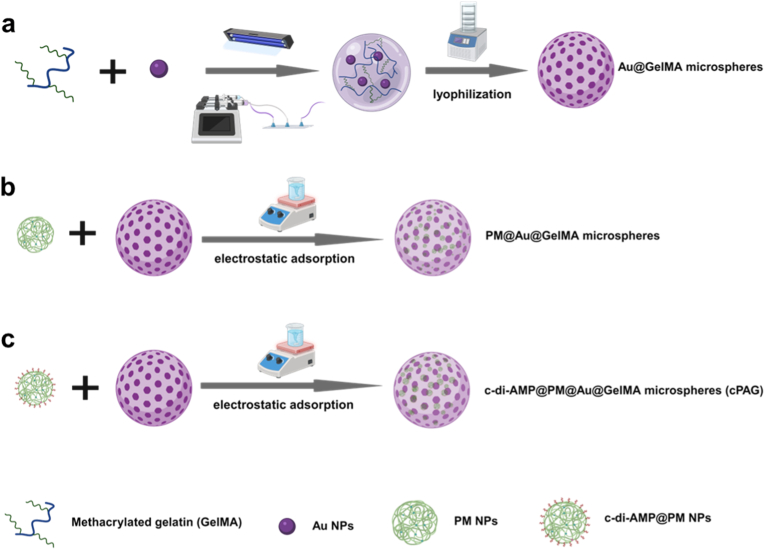
Scheme of GelMA-based microspheres fabrication. **a.** Fabrication route of Au@GelMA. Au@GelMA microspheres were fabricated using microfluidics and followed by the UV-light exposure for the photo-crosslinking. Then, the porous Au@GelMA microspheres were prepared after the lyophilization. **b.** Preparation of PM@Au@GelMA microspheres. The PM NPs were loaded on the Au@GelMA microspheres through electrostatic interaction to form the PM@Au@GelMA microspheres. **c.** Preparation of c-di-AMP@PM@Au@GelMA microspheres (cPAG). The c-di-AMP@PM NPs were also loaded on the Au@GelMA microspheres through electrostatic interaction for preparing the (cPAG). The schematic diagram was drawn using the software BioRender (https://www.biorender.com/).

Thus, here we report a two-step, nano-in-micro microsphere depot designed as a modular local platform integrating conductivity enhancement, photothermal conversion, O_2_ and ROS generation capacity, and innate immune agonist co-delivery to support a staged local treatment concept. The structural matrix consists of methacrylated-gelatin (GelMA) hydrogel microspheres embedded with gold nanoparticles (Au NPs) (Au@GelMA), which are fabricated *via* water-in-oil droplet microfluidics followed by photocrosslinking under ultraviolet (UV) exposure (Fig. 1a). Incorporation of Au NPs reduces the high bulk electrical resistance of the hydrogel matrix, and simultaneously enables tunable NIR laser-responsive heating, allowing Au@GelMA microspheres to function as a single matrix for electrical and photothermal modulation. After lyophilization, the resulting porous structure on the microspheres can absorb the manganese-oxide nanoclusters (PM NPs) or PM NPs pre-loaded with cyclic di-adenosine monophosphate (c-di-AMP@PM NPs) *via* the electrostatic interactions in the aqueous solution, forming PM@ Au@GelMA microspheres and ultimate c-di-AMP@ PM NPs@ Au@GelMA microspheres (cPAG) (Fig. 1b and c).

PM NPs exhibit catalase-like O_2_ generating capacity in H_2_O_2_ solutions and produce free radicals concurrently, and they increase inflammatory cytokine secretion, including interleukin-6 (IL-6) and tumor necrosis factor-α (TNF-α), from RAW264.7 macrophages *in vitro*. Co-delivery of c-di-AMP with PM NPs increases interferon-β (IFN-β) secretion in conditioned medium assays, consistent with induction of a type I interferon (IFN) response, although direct stimulator of interferon genes (STING) pathway protein activation was not assessed. We apply cPAG depots in a staged regimen aligned with local treatment workflow: (1) perioperative intratumoral administration to support IRE in the residual tumor setting, and (2) a second administration on postoperative 5^th^ day (*i.e.*, day 15 in whole study window) followed by NIR-laser irradiation to apply PTT. Moreover, the wet-adhesion property of the depot matrix material, GelMA, allows longtime residence at the resection interface on the remaining tumor tissue, while its enzymatic degradability supports cPAG depots disintegration and clearance within weeks in the biological environment.

Overall, this work establishes a nano-in-micro depot strategy that assists multiple local treatment-enabling functions within an injectable microsphere platform and evaluates its performance in a TNBC residual tumor model. By combining conductivity modulation, photothermal conversion, catalytic redox activity, and immune agonist co-delivery in a single locally administrated format, cPAG offers a materials framework to support combined intervention strategies for postoperative control of aggressive TNBC tumors.

## 2 Results and discussion

### 2.1 Electrical resistance of hydrogels with and without Au NPs

To verify whether the incorporation of Au NPs can lower the high-electrical resistance hydrogels, a multimeter was used to measure the bulk resistance of the prepared GelMA-based hydrogels. As shown in Fig. S1a, after incorporating Au NPs inside the GelMA hydrogels, the resistance of the hydrogel statistically significantly decreased, from 2.54 MΩ of GelMA hydrogel to 0.17 MΩ of Au@GelMA hydrogel, which could be attributed to the reported electron-conduction pathways created by the formed metallic network [[29], [30], [31]]. Moreover, the resistance decrease trend reproduced in methacrylated hyaluronic acid (HAMA)-based hydrogels once again, from 3.03 MΩ of HAMA hydrogel to 0.17 MΩ of Au@HAMA hydrogel. These results indicate that Au NPs loading increases effective electronic conductivity. Since the microspheres are microscale cross-linked hydrogel particles [32,33], Au@GelMA microspheres with enhance conductivity provide materials basis for enhanced IRE efficacy using Nanoknife on TNBC solid tumor.

### 2.2 Morphology and photothermal characteristics of Au@GelMA microspheres

**Fig. 2:**
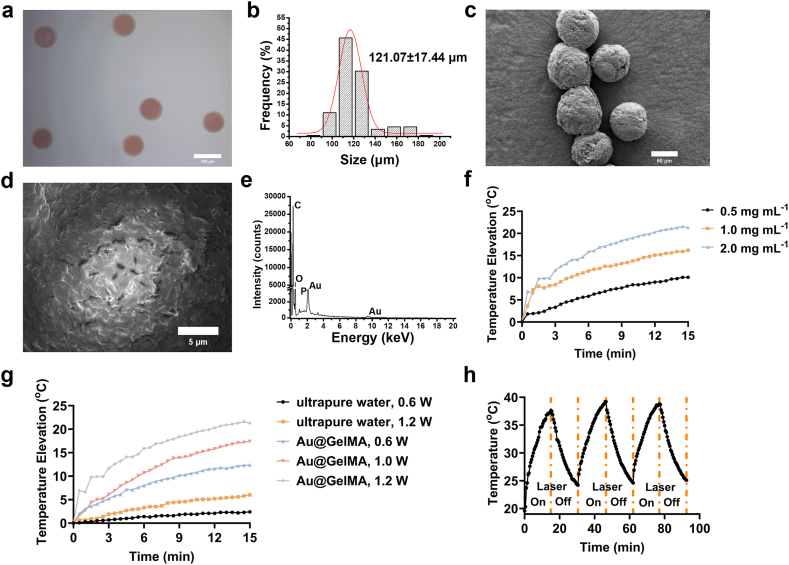
Morphological and elemental profile and photothermal effect of the Au@GelMA microspheres. **a.** Optical microscopy image of Au@GelMA microspheres. Scale bar: 150 μm. **b.** Size distribution of Au@GelMA microspheres. **c.** SEM image of Au@GelMA microsphere. Scale bar: 50 μm. **d.** SEM/EDX image of Au@GelMA microsphere surface. Scale bar: 5 μm. **e.** EDX spectrum of d. **f.** Temperature elevation of Au@GelMA microspheres with different concentrations under 808 nm laser irradiation with output power of 1.2 W. **g.** Temperature elevation of Au@GelMA microspheres (2.0 mg mL^−1^) and ultrapure water under 808 nm laser irradiation with different output powers. **H.** Temperature elevation of Au@GelMA microspheres (2.0 mg mL^−1^) with three cycles of 808 nm laser ON and 808 nm laser OFF.

Au@GelMA microspheres were produced *via* droplet microfluidics followed by photocrosslinking under UV exposure (Fig. 1a). Optical microscopy image of Au@GelMA microspheres confirmed their uniform spherical morphology when dispersed in ultrapure water (Fig. 2a), and the images-based sizing (n = 182) showed that the mean diameter of the Au@GelMA microspheres is 121.07 ± 17.44 μm (Fig. 2b), which can be easily injected with syringe needle greater than or equal to 22 G (with inner diameter ≈ 410 μm), *e.g.*, 18 G, for minimally invasive administration [34]. Scanning electron microscopy (SEM) image of the hydrated Au@GelMA microspheres also confirms the morphological uniformity, but with a shrunken size (∼70 μm) after lyophilization (Fig. 2c). These results represent an 80.64 % volume shrinkage after dehydration, demonstrating over 80 % water content in the swollen state [35]. Furthermore, SEM image with high magnification of the microsphere revealed the porous surface after the lyophilization (Fig. 2d), providing additional adsorption sites for the subsequent NPs loading. Next, the energy-dispersive X-ray spectroscopy (EDX) spectrum at the microsphere's surface exhibited distinct Au signals, confirming the incorporation of Au NPs.

Then, to quantify the photothermal capacity of the Au@GelMA microspheres endowed by Au NPs incorporation, a thermal camera was used to record the temperature change of the Au@GelMA suspensions under NIR (808 nm) laser irradiation. As shown in Fig. 2f, g and Fig. S2, the photothermal effect of the Au@GelMA microspheres is dependent on laser output power and the microspheres concentration. After 15 min of irradiation at a power of 1.20 W, the temperature of Au@GelMA microspheres of 2 mg mL^−1^ reached by 21.30 °C. Meanwhile, an increase of 16.20 °C was reached at 1 mg mL^−1^, and it was 10.10 °C at 0.5 mg mL^−1^. In contrast, the temperature of ultrapure water at same condition only increased by 6 °C. Under 0.6 W for 15 min, the temperature elevation of Au@GelMA (2 mg mL^−1^) reached to 12.4 °C, 10 °C higher than that of ultrapure water at same condition. Also, during three cycles of 15 min laser on (heating phase) and following 15 min laser off (cooling phase), the temperature rise curves and temperature drop curves of Au@GelMA microspheres (2 mg mL^−1^, maximum testing concentration) in three cycles were nearly overlapping, showing very stable photothermal effect (Fig. 2h and Fig. S3).

### 2.3 In vitro photothermal ablation and macrophages stimulation

**Fig. 3:**
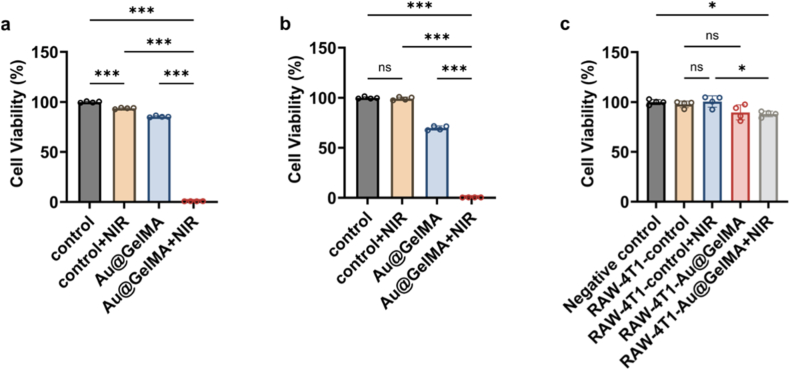
*In vitro* anti-cancer effect of Au@GelMA microspheres. **a.** 0 h cell viability of 4T1 cells after being treated with Au@GelMA microspheres (2.0 mg mL^−1^) with or without 808 nm laser exposure (1.2 W, 10 min) (n = 4). **b.** 24 h cell viability of 4T1 cells after being treated with Au@GelMA microspheres (2.0 mg mL^−1^) with or without 808 nm laser exposure (1.2 W, 10 min) (n = 4). **c.** Cell viability of 4T1 cells in culture with different stimulated RAW cells culture medium (n = 4). The error bars are based on standard errors of the mean and statistical significance was determined by ANOVA. (∗∗∗*P* < 0.001, ∗∗*P* < 0.01, or ns *P* > 0.05).

Based on clarified photothermal properties, to achieve the best treatment effect of PTT, 2 mg mL^−1^ microspheres and 1.2 W power were determined for the following anti-cancer experiments. As shown in Fig. 3a, immediately (0 h) after 10 min 808 nm laser irradiation on the 4T1 cancer cells, the cell viability in the Au@GelMA + NIR group was the lowest, down to 0.73 %, statistically significantly lower than that of Au@GelMA (85.37 %), and that of NIR (93.01 %), demonstrating excellent and immediately effective therapeutic efficacy of the PTT-mediated by the Au@GelMA microspheres. To verify whether this ablation on 4T1 cells is devastating, the *in vitro* experiment was repeated, but with prolonged incubation time after irradiation. At 24 h post-irradiation, 4T1 cancer cell viability was assessed again. As shown in Fig. 3b, the Au@GelMA combined with 808 nm laser irradiation still yielded the lowest cell viability, 0.42 %, even lower than the 0 h result, indicating durable ablation without measurable regrowth. The 24 h cell viability in Au@GelMA was 69.78 %, also lower than the 0 h result. This can be attributed to the Au NPs coated with branched polyethyleneimine (PEI, with molecular weight (MW): ∼25,000) [[36], [37], [38], [39]] from degradable GelMA microspheres. In contrast, the cell viability of NIR only increased when compared with that of 0 h, up to 99.24 %, underscoring the necessity of photothermal transducer.

To verify whether the Au@GelMA-based PTT on cancer cells can effectively stimulate the immune cells for activating anti-cancer effect, the culture medium supernatant collected from *in vitro* Au@GelMA-mediated PTT anti-cancer experiments were incubated with RAW 264.7 cells, a macrophage cell line. As the functional marker of pro-inflammatory activation of macrophages [[40], [41], [42]], the IL-6 in the RAW cells culture medium was measured. After 48 h co-incubation, the IL-6 secretion across different treatment groups, *e.g.*, 9.59 pg mL^−1^ in Au@GelMA combined with NIR related group, but without significant differences when compared with negative control group (Fig. S4). Nevertheless, the activated RAW 264.7 cells’ medium still modestly suppressed seeded 4T1 cells proliferation. 24 h after incubation, the cell viability in the Au@GelMA + NIR related group was the lowest, 88.05 %, statistically significantly lower than that in NIR related group, 100.60 % (Fig. 3c). The *in vitro* results indicate that Au@GelMA microspheres-mediated potent PTT can effectively ablate TNBC cells, but with insufficient subsequent immune cell stimulation. Therefore, the addition of an immune stimulatory enhancer is needed for better immune cells activation, such as macrophage pro-inflammatory polarization, and the corresponding anti-cancer immune response.

The IRE efficacy against TNBC cells was also tested with GelMA microspheres or Au@GelMA microspheres. As shown in Fig. S5, Au@GelMA combined with IRE therapy through a Nanoknife system yielded the lowest cell viability, outperforming that of GelMA microspheres combined with IRE, achieving the best 4T1 cells inhibition efficiency. This result is consistent with impedance reduction by Au NPs incorporation.

### 2.4 Characterization of PM NPs and PM@Au@GelMA microspheres

Free Manganese (Mn) ions, *e.g.*, Mn^2+^, have been reported to be strong immunostimulatory and act as an innate immune adjuvant [[43], [44], [45]], such as activating innate immune cells to boost antitumor immunity. Therefore, manganese oxide (MnO_x_) NPs-based nanoplatforms can act as nanoscale Mn-delivery immune adjuvant for activating macrophages, amplifying pro-inflammation or antitumor pathways, such as Toll-like receptor 4 (TLR-4) pathway [46], or triggering STING-driven interferon signals in TME [47]. In addition, MnO_x_ NPs, like MnO_2_ NPs, can act as catalase-mimicking nanozymes that can consume excessive H_2_O_2_ in acidic TME to generate O_2_ [48,49] and ROS [50,51] for hypoxia alleviation and sensitizing tumor to subsequent local therapies [52,53]. Thus, MnO_x_ NPs were chosen as the immunostimulatory adjuvant to be loaded on the Au@GelMA microspheres. Considering that the surface of GelMA is negatively charged around neutral pH or at normal physiological pH [54,55], and the simplicity of electrostatic adsorption, we prepared a MnO_x_ nanoflower using branched PEI (MW:∼25,000) as a cationic ligand, denoted PM NPs.

**Fig. 4:**
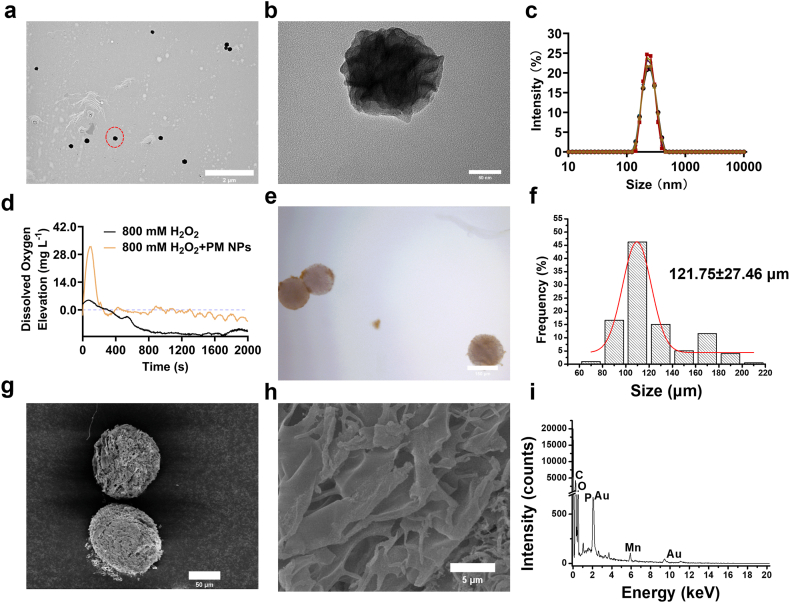
Morphological and elemental profile the PM NPs and PM@Au@GelMA microspheres. **a.** Representative TEM image of PM NPs. Scale bar: 2 μm. **b.** Higher magnification TEM image of marked PM NP with a red dashed circle in a. Scale bar: 50 nm. **c.** DLS plots of the PM NPs (n = 3). **D.** O_2_ generation profile of PM NPs (0.5 mg mL^−1^) incubated with H_2_O_2_ (800 mM). **e.** Optical microscopy image of PM@Au@GelMA microspheres. Scale bar: 150 μm. **f.** Size distribution of PM@Au@GelMA microspheres obtained from 199 particles. **g.** Representative SEM image of PM@Au@GelMA microspheres. Scale bar: 50 μm. **h.** Representative of SEM image of PM@Au@GelMA microspheres surface. Scale bar: 5 μm. **i.** EDX spectrum of h.

Transmission electron microscopes (TEM) images show that the morphology of PM NPs was very uniform (Fig. 4a), and the shape of individual PM NP was near-spherical (Fig. 4b), with ∼100 nm diameter. The size distribution of hydrated PM NPs was also narrow (Fig. 4c). Cryo-TEM image of the PM NPs further confirms the morphological uniformity and native nanoflower-like appearance in the native aqueous state without drying artifacts (Fig. S6a). EDX revealed the uniform spatial distribution of Mn and O elements in the dried PM NPs, even within individual particles (Fig. S6b–d). Additionally, the Fourier transform infrared spectroscopy (FTIR) spectra identified PEI signatures within PM NPs (Fig. S7). For example, the bands at 1651 cm^−1^ and 1593 cm^−1^, separately indicate the bending vibration of the nitrogen-hydrogen bonds (N-H) in primary and secondary amines. For another example, the bands at 2932 cm^−1^ and 1455 cm^−1^, separately, indicate the stretching vibration and in-plane bending of methylene groups (-CH_2_).

Catalase-like activity was evaluated by dissolved O_2_. As shown in Fig. 4d, there was hardly O_2_ generation in the H_2_O_2_ aqueous solution (800 mM), but in contrast, after dispersed PM NPs (0.5 mg mL^−1^) in H_2_O_2_ aqueous solution (800 mM), substantial generated O_2_ was detected within a short period of time. Moreover, this measurement was repeated in H_2_O_2_ solution in a much lower concentration (5 mM). Enhanced O_2_ generation was detected with the of PM NPs (Fig. S8), confirming the excellent catalytic capacity of the PM NPs on the H_2_O_2_. The generated ROS *via* Mn ion-mediated Fenton-like chemistry [56], especially the free radicals during the catalysis, such as highly cytotoxic hydroxyl radicals (·OH) [53], can vulnerate tumor tissues to local ablation therapy [57,58]. As shown in Fig. S9, with fluorescent nanodiamond relaxometry [59], the T1 relaxation time of the diamonds was statistically significantly shortened in H_2_O_2_ solution (0.979 mM) with the presence of PM NPs, showing much more generation of free radicals-driven by the PM NPs when compared with H_2_O_2_ solution alone. The PM NPs were then loaded on the Au@GelMA microspheres through electrostatic interactions (Fig. 1b) to form PM@Au@GelMA microspheres. The hydrated PM@Au@GelMA microspheres remained a uniform spherical morphology (Fig. 4e). After particle size statistics (n = 199), the average size of hydrated PM@Au@GelMA microspheres was 121.75 ± 27.46 μm, with relatively narrow size distribution (Fig. 4f), like Au@GelMA microspheres. SEM images of dehydrated PM@Au@GelMA also showed the similar spherical appearance (Fig. 4g) when compared with Au@GelMA (Fig. 2c), indicating that the NPs loading process hardly change the main structure and morphological profile of the microspheres. Then, higher magnification SEM images of two kinds of microspheres surface demonstrate a roughened surface on the PM@Au@GelMA microspheres due to the observed PM NPs coverage (Fig. S10a and b). The following EDX scanning on the PM@Au@GelMA surface (Fig. 4h) showed co-localized Mn and Au elements (Fig. 4i), indicating the successful loading of PM NPs *via* electrostatic adsorption.

### 2.5 In vitro macrophages activation and anti-cancer effect

**Fig. 5:**
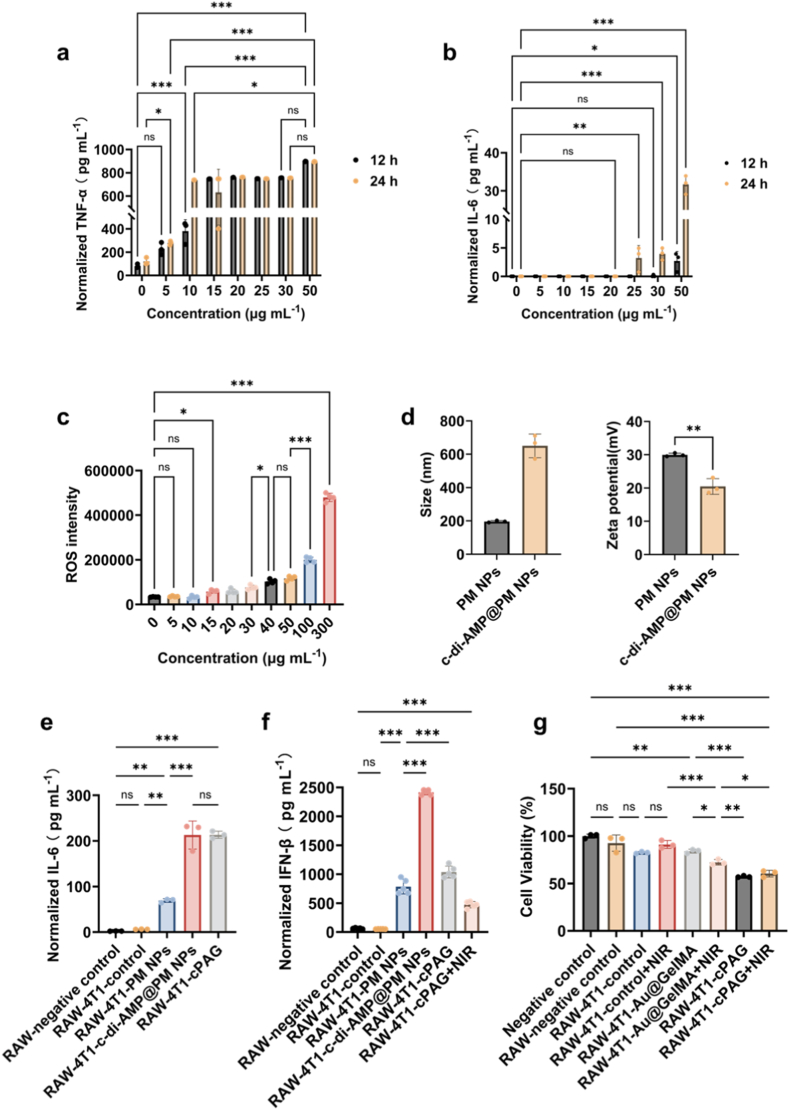
*In vitro* inflammatory response activation and anticancer ability of PM NPs, c-di-AMP@PM NPs, and cPAG depots. **a.** Normalized IL-6 secretion from RAW cells after incubation with PM NPs of different concentrations for 12 h and 24 h (n = 3). **b.** Normalized TNF-α secretion from RAW cells after incubation with PM NPs of different concentrations for 12 h and 24 h (n = 3). **c.** ROS signals of 4T1 cancer cells after being treated with different concentrations PM NPs for 24 h (n = 4). **d.** Average size and zeta potential of PM NPs and c-di-AMP@PM NPs (n = 3). **e.** Normalized IL-6 secretion of RAW cells in culture with supernatants collected from differently treated 4T1 cells (n = 3). **f.** Normalized IFN-β secretion of RAW cells in culture with supernatants collected from differently treated 4T1 cells (n = 5). **g.** Cell viability of 4T1 cells after being cultured with supernatants collected from different RAW cells, which had been stimulated by supernatants collected from differently treated 4T1 cells (n = 3). The error bars are based on standard errors of the mean and statistical significance was determined by ANOVA. (∗∗∗*P* < 0.001, ∗∗*P* < 0.01, or ns *P* > 0.05).

To clarify the pro-inflammation and anti-cancer activation capacity of the PM NPs, macrophages were incubated with the NPs for *in vitro* stimulation experiments. After 24 h co-incubation, the cell viability of the macrophages incubated with PM NPs of different concentrations (0–50 μg mL^−1^) was above 100 % (Fig. S11), indicating no overt cytotoxicity within this concentration range. Next, cytokines associated with inflammatory activation in culture supernatants, including IL-6 and TNF-α, were quantified after macrophages incubation with PM NPs [40,60,61]. As shown in Fig. 5a, the stimulated release of TNF-α by PM NPs rose at lower thresholds. When compared with that in control group (0 μg mL^−1^), even only after 12 h co-incubation and with the lowest concentration, 5 μg mL^−1^, the TNF-α secretion increased, and with the higher concentration, the higher the secretion of TNF-α. As shown in Fig. 5b, PM NPs increased IL-6 secretion in a concentration-dependent and time-dependent manner, with more pronounced increase after 24 h than 12 h. These cytokines readouts suggest that PM NPs can promote an inflammatory activation profile in RAW264.7 cells *in vitro*, consistent with reported nuclear factor kappa-B (NF-κB) engagement [60,62]. However, definite M1/M2 phenotyping (*e.g.*, CD86/CD206 co-expression) was not performed in this study.

Next, PM NPs at different concentrations were incubated with 4T1 cancer cells for intracellular ROS detection. As shown in Fig. 5c, after 24 h co-incubation, PM NPs with concentrations higher than or equal to 15 μg mL^−1^ statistically significantly rose the intracellular ROS in 4T1 cells, with NPs concentration-dependently. This indicates that PM NPs are catalytically active inside 4T1 cells, consuming intracellular peroxides into ROS *via* Fenton-like reactions, and thereby imposing oxidative stress on cancer cells.

Mn^2+^ ions released from MnO_x_ NPs can potentiate the cyclic GMP-AMP synthase-STING pathway for antitumor immunity [47]. However, Mn^2+^ ions cannot ensure the STING agonism in cells because STING still needs to be triggered [63]. Loading a STING agonist, such as c-di-AMP, on MnO_x_ NPs can provide a direct ligand for STING triggering and amplify the IFN production, especially the IFN-β with anti-tumor effect [64,65]. Anionic c-di-AMP [63,66] were adsorbed on the PM NPs with positively charged surfaces *to* prepare c-di-AMP@PM NPs [[67], [68], [69], [70]]. The c-di-AMP@PM NPs were nearly three times larger than PM NPs in size, detected by dynamic light scattering (DLS) (Fig. 5d), with 195.83 ± 5.80 nm of PM NPs while 650.13 ± 71.12 nm of c-di-AMP@PM NPs. This can be attributed to the aggregation caused by decreased surface charge after loading the c-di-AMP on the surface [71,72] (Fig. 5d). Based on the linear relationship between c-di-AMP concentration and adsorption characteristic peak (∼258 nm) of c-di-AMP solutions using UV–visible spectroscopy (Fig. S12), the calculated encapsulation efficiency and drug loading efficiency of c-di-AMP were separately 86.46 % and 7.96 %.

Afterwards, the cytotoxicity of the new prepared c-di-AMP@PM NPs to macrophages were studied. Like what we observed for PM NPs, after 24 h co-incubation of RAW264.7 cells and c-di-AMP@PM NPs, negligible cytotoxicity was observed within the tested concentration range, from 0 to 50 μg mL^−1^ (Fig. S13).

Next, as shown in Fig. 1c, the ultimate nano-in-micro microsphere depot, cPAG, was assembled *via* electrostatic interactions. To assess macrophages responses in a cancer cells-conditioned context, RAW264.7 cells were incubated with supernatants derived from different 4T1 cultures, including cPAG-related conditions. As shown in Fig. 5e, the IL-6 level in c-di-AMP@PM NPs group and cPAG group both were higher than other groups. Further, the IFN-β as a IFN readout in RAW264.7 cells culture medium was measured [[73], [74], [75], [76], [77]]. As shown in Fig. 5f and Fig. S14, the 4T1 cells samples treated with PM NPs, c-di-AMP@PM NPs, or cPAG all effectively increased IFN-β secretion compared with comparison groups. The weaker cytokine stimulation in cPAG combined with PTT could be attributed to excessive thermal stress can reduce immunogenic cell death signal *in vitro*, further decreasing the immune signaling [78]. The results are consistent with induction of an IFN response *in vitro*, although direct STING pathway activation (*e.g.*, STTING, TBK1 or IRF3 phosphorylation) was not examined. To verify whether the pro-inflammatory capacity combined with the microsphere-based PTT can suppress 4T1 proliferation, new seeded 4T1 cells were incubated with supernatants from of RAW 264.7 cells culture medium after different treatments. After 24 h, the cell viability in cPAG-derived groups, including cPAG alone and cPAG combined with NIR, were statistically significantly lower than other groups (Fig. 5g and Fig. S15), demonstrating that cPAG-associated macrophage activation produced the strongest macrophages-mediated secondary growth inhibition *in vitro*. Moreover, the macrophages stimulated with single microsphere-PTT exhibited TNBC inhibition ability, but the inhibition effect was lower than the cPAG groups, which can be attributed to the latter depot's capacity to trigger the STING pathway. Notably, thermal irradiation did not uniformly increase cytokine outputs across all readouts, suggesting that heating parameters and secretion kinetics may influence macrophage response in conditioned-medium formats.

### 2.6 In vivo anti-tumor efficacy

**Fig. 6:**
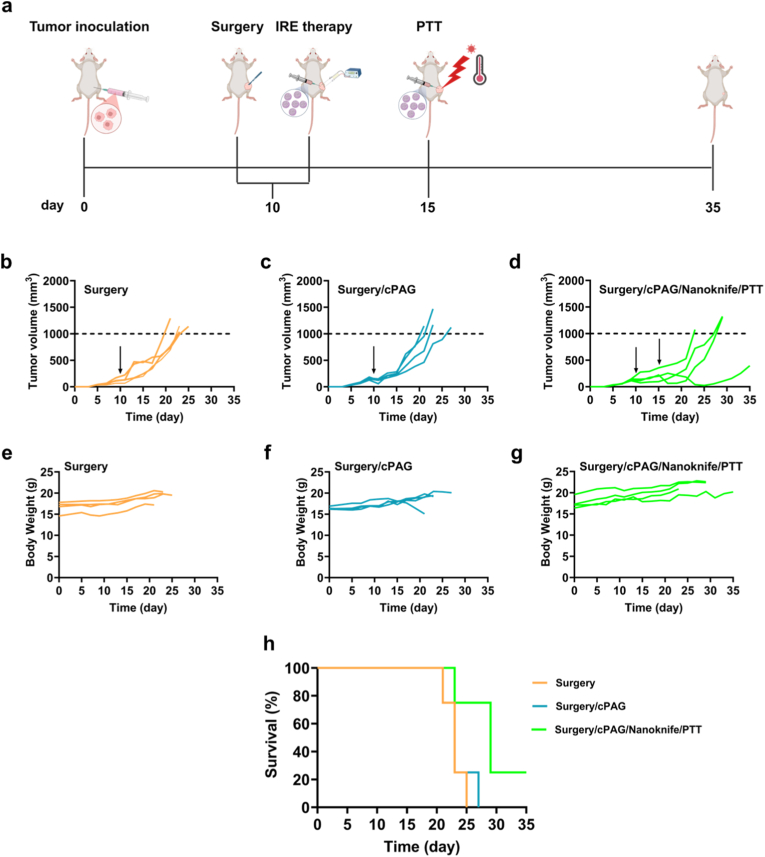
*In vivo* anti-tumor effect of cPAG combined with surgery, Nanoknife and PTT. **a.** Schematic diagram of the cPAG microsphere-based local comprehensive treatment. **b.** Individual tumor growth curves of 4T1 tumors of the Surgery group (black arrow pointing the date of surgical day, 10th day) (n = 4). **c.** Individual tumor growth curves of 4T1 tumors of Surgery/cPAG group (black arrow pointing the date of surgical day, 10th day) (n = 4). **d.** Individual tumor growth curves of 4T1 tumors of Surgery/cPAG/Nanoknife (left black arrow pointing the date of surgery combined with Nanoknife, 10th day) and afterwards PTT (right black arrow pointing the date of PTT day, 15th day) (n = 4). **e.** Individual body weight record of the Surgery group (n = 4). **f.** Individual body weight record of the Surgery/cPAG group (n = 4). **g.** Individual body weight record of Surgery/cPAG/Nanoknife/PTT group (n = 4). **h.** Survival curves of the tumor-bearing mice in different treatments group (n = 4). The Log-rank (Mantel-Cox) test for survival analysis, in comparison of HTAI + NIR group and other groups).

A staged, local multimodal treatment regimen, was evaluated *in vivo* using 4T1 tumor-bearing mice for its anti-tumor efficacy. When the average tumor volume reached approximately 130 mm^3^, the tumor-bearing mice underwent partial tumor resection to establish a residual tumor setting. Immediately after surgery, mice received intratumoral injection of cPAG microspheres and IRE therapy using Nanoknife system. On day 15, a second intratumoral microspheres injection, followed by 808 nm laser irradiation to apply PTT were performed (Fig. 6a).

As shown in Fig. 6b, in the Surgery group, after surgical treatment alone, the 4T1 tumor recurred very rapidly due to the residual tumor tissue, consistent with aggressive local recurrence from residual tumor. Next, in the Surgery/cPAG group (Fig. 6c), the cPAG microspheres injected post-surgery effectively slowed the early recurrence rate of the residual tumors, but significant acceleration in tumor growth occurred subsequently in Surgery/cPAG. In contrast, in the Surgery/cPAG/Nanoknife/PTT group, residual tumor regrowth was more strongly suppressed compared with another two groups during the study window (Fig. 6d). On day 11, one day post-surgery, the average tumor volume in each group was similar, with 138.56 ± 64.76 mm^3^ in Surgery, 113.53 ± 38.36 mm^3^ in Surgery/cPAG, and 150.89 ± 87.34 mm^3^ in Surgery/cPAG/Nanoknife/PTT. On day 15, although the average tumor size in Surgery/cPAG/Nanoknife/PTT was 217.30 ± 106.74 mm^3^, smaller than 345.57 ± 145.07 mm^3^ in Surgery and 243.27 ± 29.86 mm^3^ in Surgery/cPAG, but with no significant difference. On day 19, the last time point with all tumors below 1000 mm^3^, the average tumor volume in Surgery/cPAG/Nanoknife/PTT was the smallest, with 240.01 + 155.36 mm^3^, statistically significantly lower than 635.38 ± 148.35 mm^3^ in Surgery and 621.60 ± 184.57 mm^3^ in Surgery/cPAG (Fig. S16a–c). These multiple time-points’ tumor volumes indicate that cPAG assisting staged local treatment regimen can effectively inhibit TNBC tumor regrowth. The body weight of tumor-bearing mice remained stable across groups (Fig. 6e–g), indicating tolerability under the applied local treatment conditions. However, comprehensive organ histology and serum biochemistry were not assessed in this study.

Survival was monitored until tumors reached the humanitarian endpoint volume threshold. As shown in Fig. 6h, tumor-bearing mice receiving the staged cPAG-based treatment showed prolonged survival, when compared with another two groups. Survival curves were generated using the Kaplan-Meier method with long-rank test. Given the small cohort size (n = 4), statistical hypothesis testing of survival differences was not emphasized.

Overall, these data demonstrate that staged multimodal regimen incorporating cPAG with IRE and delayed PTT produced the strongest suppression of postoperative local tumor regrowth among the tested groups in this residual tumor model.

## 3 Conclusion

Incorporation of Au NPs within the GelMA hydrogels reduced bulk electrical resistance and enabled photothermal conversion, supporting the fabrication of injectable Au@GelMA microspheres with stable NIR laser-responsive heating. The lyophilization-caused porous surfaces on the microspheres facilitated secondary therapeutic agent loading of the PM NPs and c-di-AMP *via* electrostatic interactions, forming a nano-in-micro monodispersed cPAG depot. PM NPs showed excellent catalase-like capacity for O_2_ generation and produced free radicals in H_2_O_2_-rich solutions. Furthermore, PM increased pro-inflammatory cytokines secretion (IL-6 and TNF-α) from RAW264.7 macrophages *in vitro* and elevated intracellular ROS in 4T1 cells. Co-loading c-di-AMP, as STING agonist, onto PM NPs increased IFN-βsecretion in RAW264.7 conditioned-medium assays, consistent with induction of IFN response. *In vivo*, in a partial 4T1 model, a staged local regimen (with surgery assisted with cPAG and IRE therapy, followed by cPAG synergized with PTT) produced the most pronounced inhibition of postoperative local tumor regrowth among the tested groups, with stable body weight over the study period.

This study primarily establishes a nano-in-micro design and demonstrates improved control of postoperative local tumor regrowth in the tested *in vivo* groups. However, several mechanistic and translational evaluations were not included, mainly including direct *in vivo* hypoxia/ROS mapping, definitive macrophages phenotyping by surface expression markers, STING pathway protein activation assay, stepwise *in vivo* comparison groups (such as Surgery/IRE, or Surgery/PTT), *in vivo* anti-metastasis assessment, and comprehensive systemic safety panels. These will be important directions for future work.

Overall, cPAG illustrates a modular nano-in-micro depot design that integrates conductivity tuning, photothermal conversion, and immune agonist co-delivery to support combined local intervention strategies for aggressive TNBC solid tumors.

## 4 Materials and methods

### 4.1 Materials

The main used chemical reagents, including HAuCl_4_, branched PEI (MW:∼25,000), NaBH_4_, gelatin, methacrylic anhydride (MA), Na_2_CO_3_, NaHCO_3_, phosphate-buffered saline (PBS), dialysis bag, lithium phenyl-2,4,6-trimethylbenzoylphosphinate (LAP), liquid paraffin, squalane Monostearate (Span-80), diethyl ether (≥99.9 %), KMnO_4_, c-di-AMP sodium salt, hyaluronic acid (HA), were purchased from Merck Chemical Technology (Shanghai) Co., Ltd., China. The 2′,7′-Dichlorodihydrofluorescein diacetate (DCFDA) probe was purchased from MedChemExpress LLC., USA. CellTiter-Glo™ (luminescence cell viability assay kit) was purchased from Promega Co., Ltd., China. Mouse TNF-α precoated enzyme linked immunosorbent assay (ELISA) kit (item number:1,217,202) was provided by Dakewe Biotech Co., Ltd., China. Mouse IL-6 precoated ELISA kit and mouse IFN-β precoated ELISA kit were provided by Shanghai Enzyme-linked Biotechnology Co., Ltd., China. Dulbecco's Modified Eagle's Medium (DMEM, Gibco) and fetal bovine serum (FBS, Gibco) were purchased from Thermo Fisher Scientific Inc., USA. The Fluorescent Nanodiamonds (FNDs, made from high pressure temperature synthesis, with 70 nm hydrodynamic diameter, oxygen terminated, irradiated for nitrogen vacancy center formation) used in this were purchased from Adamas Nanotechnologies (North Carolina), USA.

### 4.2 Methods

#### 4.2.1 The synthesis of Au NPs

The synthesis of Au NPs was performed as reported in our paper [79]. In brief, 30 mL HAuCl_4_ (5.40 mg mL^−1^) were slowly added into 10 mL PEI aqueous solution (5 mg mL^−1^) under magnetic stirring at speed of 1000 rpm. After 30 min, 0.9 mL NaBH_4_ solution (50.40 mg mL^−1^) were added dropwise to the reaction solution under stirring. After another 2 h, the PEI-coated Au NPs solution with deep purple-red color was collected in a dialysis bag with cut-off molecular weight of 12–14 kDa for dialysis in ultrapure water. After 2 d, the dialyzed Au NPs was taken out for 4 °C storage.

#### 4.2.2 GelMA synthesis and HAMA synthesis

GelMA: 20 g gelatin were dissolved in 200 mL carbonate buffer (pH∼9.0) containing Na_2_CO_3_ (1.71 mg mL^−1^) and NaHCO_3_ (15.46 mg mL^−1^) with magnetic stirring under 50 °C in oil bath. After the gelatin completely dissolved, 2 mL MA were slowly added into the gelatin solution with a micro-injection pump (0.20 mL min^−1^) under 50 °C in darkness. Afterwards, the solution kept stirring for another 3 h and then 100 mL PBS were added to terminate the reaction. After that, the collected reaction solution was centrifuged for 15 min at speed of 7000 rpm to remove the excessive unreacted MA and the precipitated GelMA was dialyzed in ultrapure water for 2 d under 38 °C. Then, the purified GelMA solution was lyophilized for dehydrated solidification and stored at −80 °C.

HAMA: In brief, 500 mL HA aqueous solution (20 mg mL^−1^) were reacted with dropwise-added 20 mL MA solution under stirring at room temperature. Then, 20 mL NaOH aqueous solution (5 M) were added slowly. After overnight reaction, the collected solution was dialyzed in ultrapure water for 3 d. Afterwards, the dialyzed HAMA solution was lyophilized for dehydrated solidification and stored at −80 °C.

#### 4.2.3 Au@GelMA and GelMA microspheres preparation

Au@GelMA microspheres: After dissolving 108.60 mg GelMA in 2 mL ultrapure water at 60 °C, 10.86 mg LAP were added under magnetic stirring. After LAP was dissolved, 1.62 mL Au NPs aqueous dispersion (4.47 mg mL^−1^) were added into the GelMA/LAP solution in darkness under magnetic stirring. After 2 h mixing, the 3.62 mL GelMA/LAP/Au NPs mixed solution were loaded in a syringe as “water phase” and liquid paraffin containing span-80 (5 %, w:w) in another syringe used as “oil phase” to prepare uncrosslinked Au@GelMA microspheres precursor *via* “water-in-oil” droplet microfluidics. Then, the collected Au@GelMA microsphere precursors were exposed to 385 nm light for 15 min for LAP-mediated free-radical photocrosslinking to form the Au@GelMA microspheres. After that, the crosslinked microspheres were washed with diethyl ether (≥99.9 %) three times and then were washed with ultrapure water three times for purification. Afterwards, the obtained Au@GelMA microspheres were freeze-dried to produce porous surface and then the lyophilized porous Au@GelMA microspheres were stored at −20 °C.

GelMA microspheres: In brief, 10.86 mg LAP were then added into 3.62 mL GelAM aqueous solution (30 mg mL^−1^) in darkness under magnetic stirring. After 2 h mixing, the GelMA/LAP mixed solution was used as “water phase” and liquid paraffin containing span-80 (5 %, w:w) as “oil phase” to prepare uncrosslinked GelMA microspheres precursor *via* “water-in-oil” droplet microfluidics. Then, after 15 min of UV light (385 nm) exposure and subsequent LAP-mediated photocrosslinking, GelMA microspheres were prepared. After that, the crosslinked microspheres were washed with diethyl ether (≥99.9 %) and ultrapure water for purification. Afterwards, the obtained GelMA microspheres were freeze-dried to produce porous GelMA microspheres and then were stored under −20 °C.

#### 4.2.4 MnO_x_ nanoflowers (PM NPs) fabrication

Briefly, 3 mL HAuCl_4_ were mixed with 1 mL KMnO_4,_ and then the mixed solution was added into 24 mL PEI aqueous solution (5 mg mL^−1^) under magnetic stirring at speed of 700 rpm. After 30 min, 0.90 mL NaBH_4_ (4 mg mL^−1^) were added into the reaction solution drop by drop at room temperature. After another 2 h of magnetic stirring, the reaction solution was collected in dialysis bag for overnight dialysis in ultrapure water. Then the dialyzed solution was centrifuged for 20 min (1000 rcf) three times to obtain the precipitated PM NPs.

#### 4.2.5 Fabrication of c-di-AMP@PM NPs

87.5 μL c-di-AMP aqueous solution (1 mg mL^−1^) were added into 1 mL PM NPs dispersion (0.88 μg mL^−1^) under magnetic stirring at 4 °C. After 2 h stirring, the collected solution was centrifuged three times (10,000 rcf, 10 min) to obtain the supernatants and precipitated c-di-AMP@PM NPs. The loading efficiency was quantitatively determined using Lambda 2 spectrometer (PerkinElmer, USA). The calculation shows that the relationship between the value of the characteristic peak (∼258 nm) and c-di-AMP concentration is represented by the following equation, Y = 0.15 + 0.07X, where Y represents the value of the characteristic peak (∼258 nm), and X represents the c-di-AMP concentration.

#### 4.2.6 Preparation of PM@Au@GelMA and cPAG microspheres

1 mL PM NPs aqueous dispersion (223.74 μg mL^−1^) was added into 5.6 mL GelMA dispersion (400 μg mL^−1^) for mixing under stirring at room temperature. After a while, the collected dispersion was centrifuged at 700 rpm for 10 min to obtain the precipitated PM@Au@GelMA microspheres. Like the process of PM@Au@GelMA preparation, the mixed NPs and microsphere dispersions were centrifuged at 4 °C to obtain the precipitated cPAG microspheres.

#### 4.2.7 Preparation of GelMA-based hydrogels

Briefly, with constant concentration of each component, 2 mL of GelMA/LAP solution or GelMA/LAP/Au NPs solution were separately added into one well of a 12-well plate and followed by 15 min UV light (385 nm) exposure to form crosslinked GelMA hydrogel and Au@GelMA hydrogel.

#### 4.2.8 Preparation of HAMA-based hydrogels

2 mL HAMA/LAP solution (HAMA: 20 mg mL^−1^; LAP: 2.5 mg mL^−1^) and 2 mL HAMA/LAP/Au NPs solution (HAMA: 20 mg mL^−1^; LAP: 2.5 mg mL^−1^; Au NPs: 2 mg mL^−1^) were separately added into one well of a 12-well plate and followed by 15 min UV light (385 nm) exposure to form crosslinked HAMA hydrogel and Au@HAMA hydrogel.

#### 4.2.9 Electrical resistance measurements of the hydrogels

After the hydrogels were prepared in wells, a handheld multimeter with two-probe (DL8490, Deli Group Co., Ltd., China) was used to measure the electrostatic resistance of the hydrogels. During each measurement, the distance between the two probes was kept as consistent as possible. Three sites were randomly selected for measurement on each hydrogel sample.

#### 4.2.10 Characterization of morphological profiles

The hydrated microsphere morphology observation and imaging was conducted using DMR Microscope (Leica Microsystems GmbH, USA). The SEM images and the SEM/EDX scanning of the dehydrated microspheres were obtained with Hitachi s-4800 (Hitachi, Ltd., Japan) equipped with a HORIBA EMAX mics2 (HORIBA, Ltd., Japan).

The DLS and Zeta potential of NPs were measured using Zetasizer Nano ZS 90 (Malvern Instruments Ltd., UK). The TEM images of dehydrated NPs were captured with TFS TalosF200i (Thermo Fisher Scientific Inc., USA). The cryo-TEM images of the frozen NPs were obtained using Tecnai T20 (FEI Company, USA) operating at 200 keV and following EDX was conducted with the equipped X-Max 80 detector (Oxford Instruments Ltd., UK).

#### 4.2.11 Photothermal effect determination

The Au@GelMA microsphere dispersions with different concentrations were prepared in ultrapure water. 1.5 mL of each dispersion were added in respective 2 mL vial (polypropylene). Then each sample was separately irradiated with an optical-fiber-coupled power-tunable diode laser (808 nm, PSU-III-led, Roithner Lasertechnik GmbH, Austria) for 15 min at output power of 1.2 W. Then, 1.5 mL Au@GelMA (2 mg mL^−1^) and ultrapure water (1.5 mL) were irradiated with an 808 nm laser at different output powers. The temperature change and photographic images were recorded with a FLIR E8 XT thermal camera (Teledyne FLIR LLC., USA). Next, with 15 min laser on and followed by 15 min laser off as a testing cycle, three cycles were operated for photothermal stability investigation.

#### 4.2.12 *In vitro* anti-cancer effect of Au@GelMA microspheres

About 1 × 10^4^ 4T1 cells were seeded in one well of 96-well plates and then were incubated at 37 °C with 5 % CO_2_. After 24 h, the fresh culture medium (DMEM-based, 10 % FBS (v:v), 1 % penicillin-streptomycin (v:v)) with or without Au@GelMA microspheres (2 mg mL^−1^) replaced the old culture medium and then the each well in the control + NIR and the Au@GelMA + NIR group was irradiated with 808 nm at 1.2 W output power for 10 min. Immediately after the irradiation, according to the manufacturer's provided protocol, the cells in 96-well plates were tested with CellTiter-Glo™ kits and Synergy™ H1 plate reader (BioTek Instruments, Inc., USA) to obtain 0 h cell viability post-treatment. For evaluation of prolonged anti-cancer efficacy (24 h cell viability), after the irradiation, the 96-well plates were put back in the incubator (30 °C, 5 % CO_2_) for another 24 h. Afterwards, the cell viability of the treated 4T1 cells were assessed.

#### 4.2.13 Au@GelMA microspheres-conditioned macrophages anticancer study

As for the downstream macrophages-mediated anti-cancer assessment, the experimental procedure is briefly described as follows: ∼1 × 10^4^ RAW 264.7 cells were seeded in each well of a 96-well plate and incubated in the incubator (37 °C with 5 % CO_2_) for 24 h. Then, the fresh culture medium mixed with centrifuged Au@GelMA-treated 4T1 cell medium (v:v) was used to replace the old medium of the RAW 264.7 cells. After 48 h co-incubation, the culture medium of the RAW 264.7 cells was collected and centrifuged at 2000 rpm for 20 min. The IL-6 level in the obtained supernatant samples was assessed using IL-6 precoated ELISA kit and plate reader by following the manufacturer's manual. The remaining supernatants were mixed with new culture medium (v:v) to replace the old medium of 4T1 cells seeded one day before. Then after 24 h, the cell viability of 4T1 cells was assessed with CellTiter-Glo™ kits and a plate reader.

#### 4.2.14 O_2_ generation

The 800 mM deoxygenated H_2_O_2_ solution (pH∼7.4) was mixed with or without PM NPs (0.5 mg mL^−1^) and then WTW ProfiLine Oxi 3310 dissolved oxygen-meter (Xylem Inc., USA) was used to record the O_2_ content in the solution every 10 s at room temperature. The entire measurement lasted 2000 s. Then the O_2_ generation experiment was repeated with 5 mM H_2_O_2_ solution (pH∼7.4).

#### 4.2.15 Free radical measurement

200 μL FND solution (10 μg mL^−1^) were added to each quadrant of a glass-bottom petri dish followed by drying at 65 °C. Then for T1 relaxation time measurements, two solutions were prepared: 0.979 mM H_2_O_2_ and 0.979 mM H_2_O_2_ with PM NPs (0.15 mg mL^−1^). During the measurement, the nitrogen-vacancy (NV) defects in diamond were used to measure the free radical generation as described earlier [80]. To the end, a custom-built confocal microscope with an acousto-optical modulator (model 3350-199, Gooch&Housego, UK). T1 measurements were conducted eight times per sample.

#### 4.2.16 *In vitro* PM NPs-mediated macrophages pro-inflammatory activation

In brief, ∼1 × 10^4^ RAW 264.7 cells were seeded in each well of 96-well plate and incubated at 37 °C with 5 % CO_2_ for 24 h. Afterwards, the fresh culture medium containing PM NPs with different concentrations (0, 5, 10, 15, 20, 25, 30, 50 μg mL^−1^) replaced old medium. After another 24 h co-incubation, the cell viability of macrophages was assessed. After determining the safe concentration range, the whole co-incubation experiment between PM NPs and RAW 264.7 cells was repeated twice due to different incubation time. After 12 h or 24 h, the culture medium of activated macrophages was collected and centrifuged at 2000 rpm for 20 min to obtain the supernatant samples. The IL-6 and TNF-α level were evaluated with ELISA kits separately.

#### 4.2.17 Intracellular ROS detection

About 1 × 10^4^ 4T1 cells were seeded in each well of 96-well plate and incubated at 37 °C with 5 % CO_2_ for 24 h. Afterwards, the fresh culture medium containing PM NPs with different concentrations (0, 5, 10, 15, 20, 30, 40, 50, 100, 300 μg mL^−1^) replaced old medium. After another 24 h co-incubation, the intracellular ROS were quantitatively detected using DCFDA as fluorescent probe and plate reader. In brief, after removing the old medium and washing with PBS three times, 10 μM DCFDA (in PBS) were incubated with 4T1 cells in dark for 30 min at 37 °C. Then after removal of excessive DCFDA and washing, PBS were then added into wells and followed by detection with plate reader at excitation/emission at 485/535.

#### 4.2.18 *In vitro* macrophage pro-inflammatory activation for anticancer activity

Following the protocol described above in 4.2.13, briefly, ∼1 × 10^4^ RAW 264.7 cells were seeded in each well of a 96-well plate and incubated in the incubator (at 37 °C with 5 % CO_2_) for 24 h. Then, the fresh culture medium mixed with supernatants from differently treated 4T1 cells (v:v, 1:1) was used to replace the old medium of macrophages. After 48 h co-incubation, the culture medium of the RAW 264.7 cells was collected and centrifuged. The IL-6 and IFN-β level in the supernatant were separately assessed using an ELISA kit and a plate reader. The remaining culture medium supernatants were mixed with new culture medium (v:v) to replace the old medium of 4T1 cells seeded one day before. Then after 24 h, the cell viability of 4T1 cells was assessed with CellTiter-Glo™ kits and a plate reader.

#### 4.2.19 *In vivo* antitumor efficacy

The animal experimental procedures were conducted in accordance with the “Experimental Animal Research and Use Program” of Shanghai Jiao Tong University, “Code of Practice Animal Experiments in Cancer Research” of University of Groningen and finally approved by the Animal Ethics Committee of Shanghai Junbo Biotechnology Co., Ltd. (The application files approval number are IACUC 2024-007). All Balb/c mice (female, 4–6 weeks, SPF) were purchased from Jiangsu Huachuang Xinnuo Pharmaceutical Technology Co., Ltd., China.

About 5 × 10^5^ 4T1 cells dispersed in PBS were subcutaneously injected into the lateral of right limb of Balb/c mice to inoculate the TNBC solid tumor model. The date of tumor inoculation was denoted as day 0. Then, when the average tumor size reached up ∼130 mm^3^ on day 9, the tumor-bearing mice were randomly divided into 3 groups: Surgery, Surgery/cPAG, and Surgery/cPAG/Nanoknife/PTT.

On the day of group assessment (day 10), all the anesthetic tumor-bearing mice underwent tumor resection treatment, removing half of the original tumor to create a gross residual tumor model, which has been explicitly reported in prior research [81,82]. Afterwards, the postoperative mice in the Surgery group were sutured and put back to their enclosures for observation. The postoperative mice in the Surgery/cPAG group were intratumorally injected with 50 μL cPAG (2 mg mL^−1^) and then put back to their enclosures for observation after being sutured. The postoperative mice in the Surgery/cPAG/Nanoknife/PTT group were intratumorally injected with 50 μL cPAG (2 mg mL^−1^) and followed by IRE therapy using BTX ECM 830 Electro Square Porator Electroporator as Nanoknife (Harvard Bioscience, Inc., USA) at 140 V. After that, the postoperative and post-IRE mice were sutured and put back to their enclosures for observation. On day 15, the tumor-bearing mice in the Surgery/cPAG/Nanoknife/PTT group were intratumorally injected with 50 μL cPAG (2 mg mL^−1^) again and the tumor sites were exposed upon 808 nm (1.2 W) for 5 min. From day 0, the tumor volume of each mouse was measured every other day using a vernier caliper. The tumor volume was calculated based on the following equation: Tumor volume (mm^3^) = 0.5 × tumor length × tumor width^2^. Once the tumor volume was over 1000 mm^3^, the tumor-bearing mouse was euthanized with CO_2_ and intercervical dislocation. From day 0, the body weight of each mouse was weighted every two days using an electronic scale.

## Statistical analysis

All data were presented as mean ± standard deviation (SD) unless specified otherwise. Statistical comparisons between more than two groups were determined by ANOVA or Student's t-test for comparisons between two groups, and the Log-rank (Mantel-Cox) test was used for survival curves statistical analysis. Statistically significant differences were demonstrated as different star icons (∗∗∗: P < 0.001, ∗∗: P < 0.01, ∗: P < 0.05 or Non-significance (NS): P > 0.05). Statistical analysis was performed with GraphPad Prism 10.o version and Origin Pro. 2025 version.

## CRediT authorship contribution statement

**Jiachen Li:** Conceptualization, Data curation, Formal analysis, Investigation, Methodology, Software, Visualization, Writing – original draft. **Yaping Zhuang:** Conceptualization, Formal analysis, Methodology, Software. **Huijie Han:** Formal analysis, Methodology, Software. **Yuewen Zhu:** Formal analysis, Investigation, Methodology. **Chao Lin:** Investigation, Methodology. **Rui Wang:** Investigation, Methodology. **Ana Catarina Rodrigues da Silva:** Data curation, Investigation. **Marc C.A. Stuart:** Investigation, Resources, Visualization, Writing – review & editing. **Guimei Jiang:** Investigation, Methodology. **Siyu Fan:** Investigation, Methodology. **Romana Schirhagl:** Methodology, Resources, Writing – review & editing. **Mohammad-Ali Shahbazi:** Investigation, Visualization, Writing – review & editing. **Lígia Raquel Marona Rodrigues:** Methodology, Resources. **Wenguo Cui:** Conceptualization, Funding acquisition, Methodology, Resources, Supervision, Writing – review & editing. **Hélder A. Santos:** Conceptualization, Funding acquisition, Methodology, Resources, Supervision, Writing – review & editing.

## Declaration of competing interest

The authors declare that they have no known competing financial interests or personal relationships that could have appeared to influence the work reported in this paper.

## Data Availability

Data will be made available on request.
