# Supplementary material for: Nano-in-Micro GelMA depots assist electro-thermal-immuno orchestral treatment for solid triple negative breast tumor
Source: Mater Today Bio. 2026 Jan 30;37:102848. doi: 10.1016/j.mtbio.2026.102848 (PMC12890829; doi:10.1016/j.mtbio.2026.102848)
Supplement: Multimedia component 1 [file mmc1.docx]

**SUPPORTING INFORMATION**

**Nano-in-Micro GelMA depots assist electro-thermal-immuno orchestral treatment for solid triple negative breast tumor**

Jiachen Li^1,2^, Yaping Zhuang^1^, Huijie Han^2,3^, Yuewen Zhu^2^, Chao Lin^1,4^, Rui Wang^1,5^, Ana Catarina Rodrigues da Silva^2,7,8^, Marc C. A. Stuart^6^, Guimei Jiang^1^, Siyu Fan^2^, Romana Schirhagl^2^, Mohammad-Ali Shahbazi^2^, Lígia Raquel Marona Rodrigues^7,8^, Wenguo Cui^1,^* and Hélder A. Santos^2,^*

^1^Department of Orthopaedics, Shanghai Key Laboratory for Prevention and Treatment of Bone and Joint Diseases, Shanghai Institute of Traumatology and Orthopaedics, Ruijin Hospital, Shanghai Jiao Tong University School of Medicine, 197 Ruijin 2nd Road, Shanghai 200025, P. R. China.

^2^Department of Biomaterials and Biomedical Technology, The Personalized Medicine Research Institute (PRECISION), University Medical Center Groningen, University of Groningen, 9713 AV Groningen, the Netherlands.

^3^College of Chemistry and Life Science, Beijing University of Technology, 100 Pingleyuan, Chaoyang District, Beijing 100124, China.

^4^Wuxi School of Medicine, Jiangnan University, Wuxi, Jiangsu 214000, China.

^5^The International Peace Maternity and Child Health Hospital, School of Medicine, Shanghai Jiao Tong University, Shanghai 200030, China.

^6^Groningen Biomolecular Sciences and Biotechnology Institute, Faculty of Science and Engineering, University of Groningen, Nijenborgh 7, 9747 AG Groningen, The Netherlands.

^7^CEB-Centre of Biological Engineering, Universidade do Minho, Campus de Gualtar, Braga 4710-057, Portugal

^8^LABBELS, Associate Laboratory, Braga/Guimarães, Portugal

***Corresponding authors:** wgcui@sjtu.edu.cn; h.a.santos@umcg.nl

**
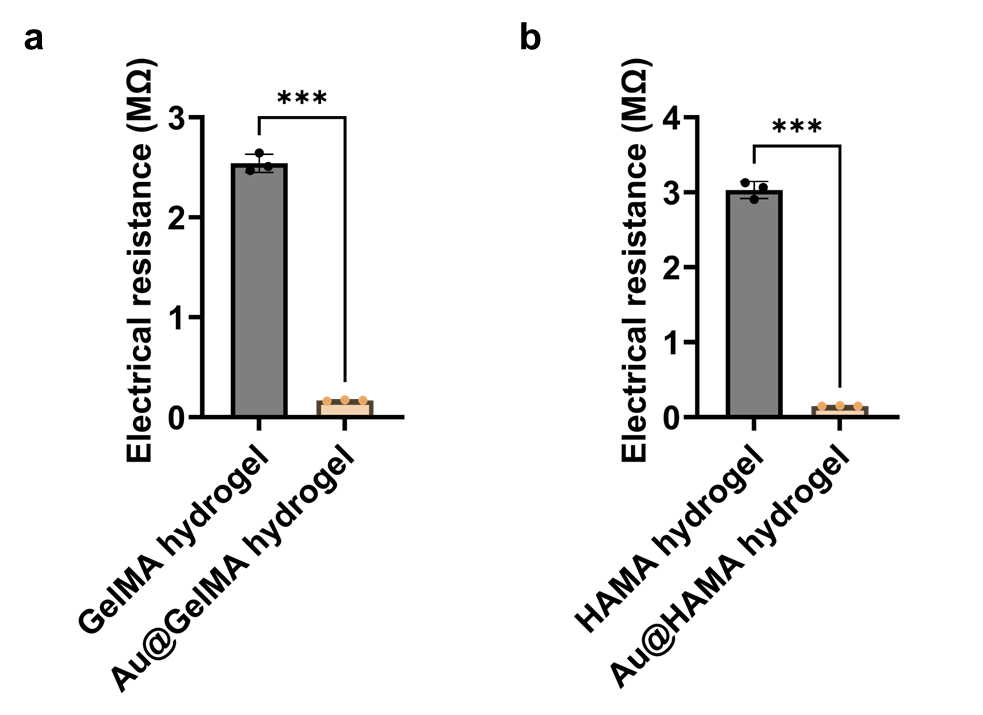
**

**Figure S1. a.** Resistance of GelMA-based hydrogel and Au NPs embedded GelMA hydrogel (n=3). **b.** Resistance of HAMA-based hydrogel and Au NPs embedded HAMA hydrogel (n=3). Error bars are based on standard errors of the mean and statistical significance was determined by ANOVA. (****P* < 0.001, ***P* < 0.01, or NS *P* > 0.05).

**
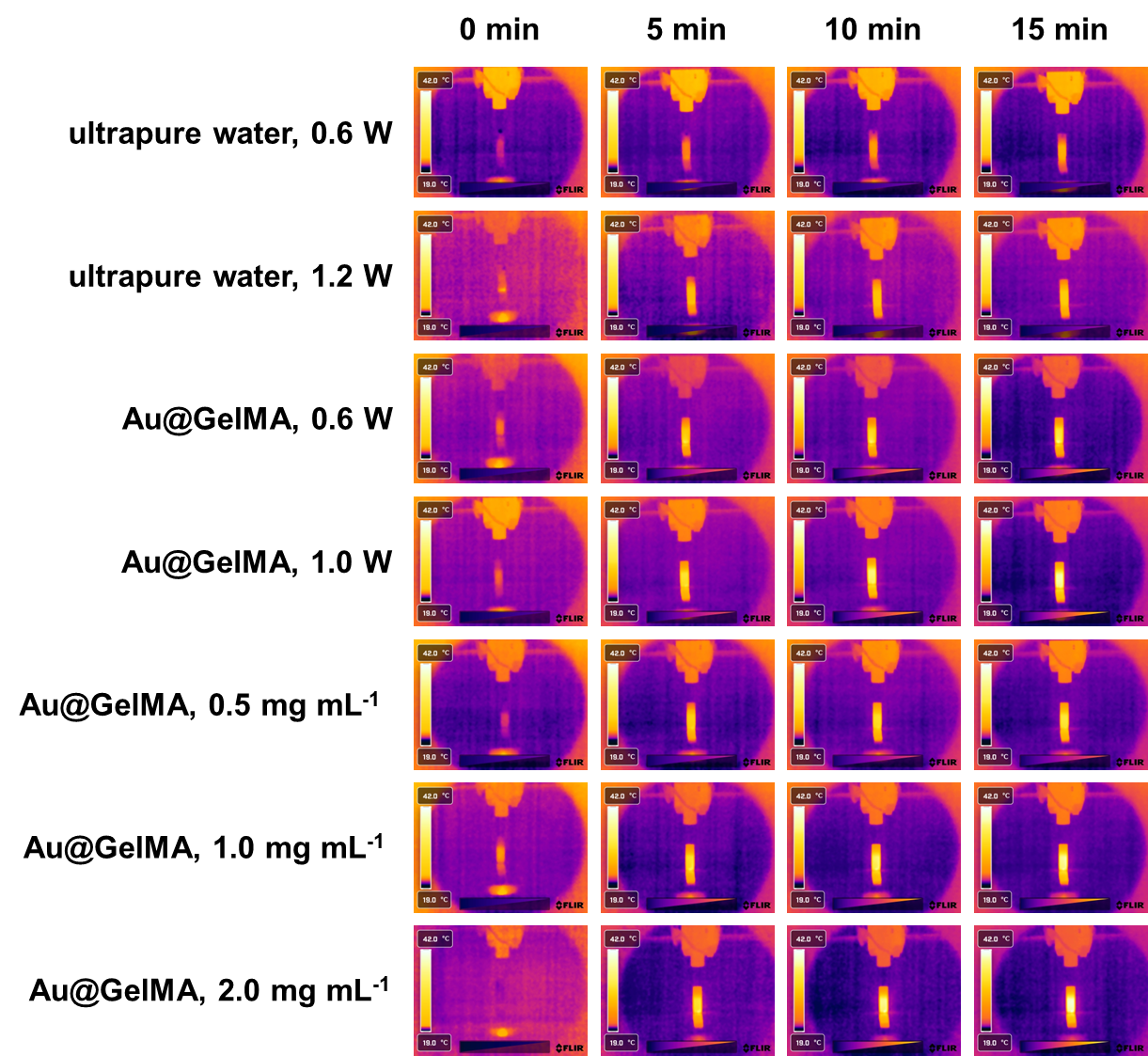
**

**Figure S2.** Photothermographic images of ultrapure water and Au@GelMA (2 mg mL^-1^) under 808 nm laser irradiation (0.6 W and 1.0 W) and the Photothermographic images of Au@GelMA (0.5 mg mL^-1^, 1.0 mg mL^-1^, 2.0 mg mL^-1^) under 808 nm laser irradiation (1.2 W).

**
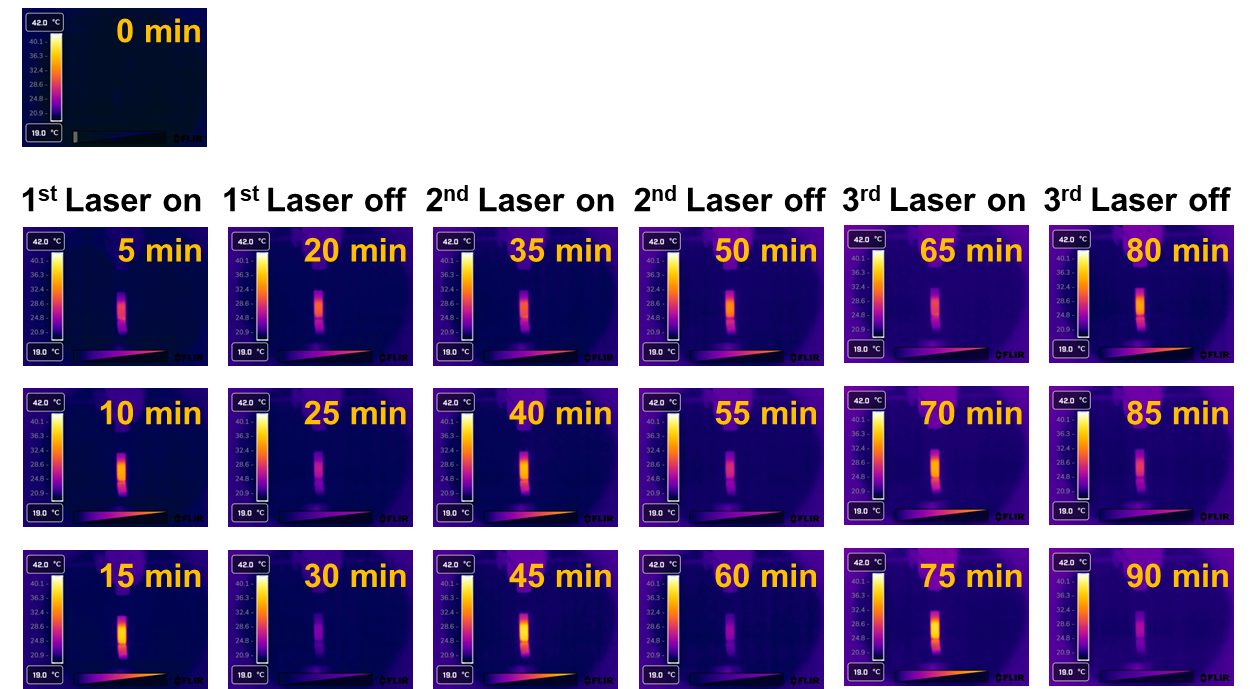
**

**Figure S3.** Photothermographic images of Au@GelMA (2 mg mL^-1^) under three cycles of 808 nm laser ON and 808 nm laser OFF.

**
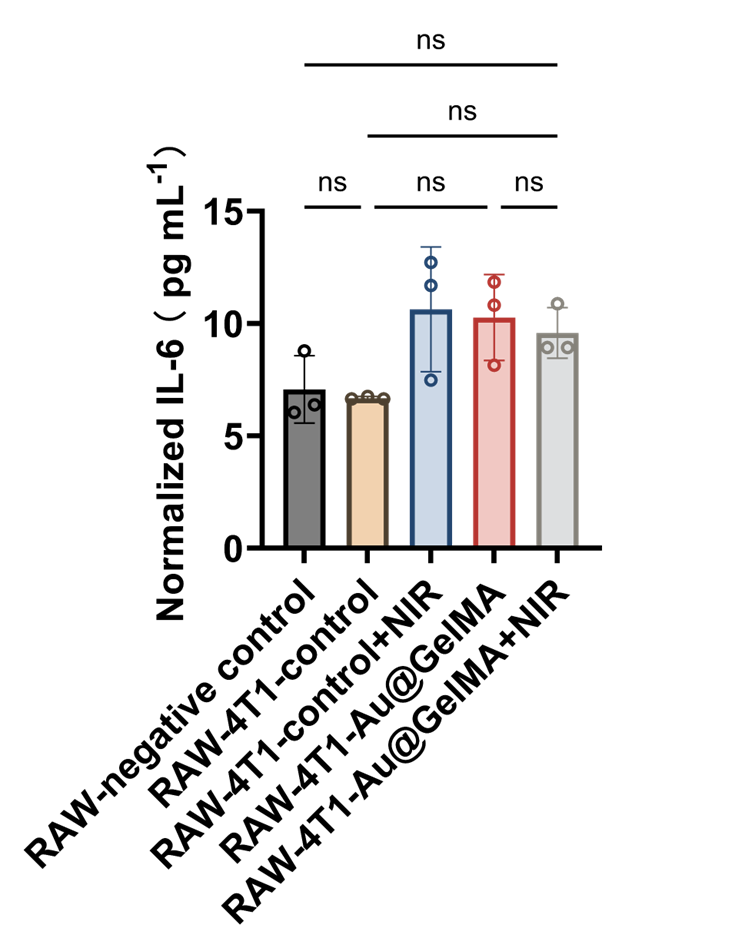
**

**Figure S4.** Normalized IL-6 secretion of RAW cells in culture with supernatant collected from differently treated 4T1 cells (n=3). Error bars are based on standard errors of the mean and statistical significance was determined by ANOVA. (****P* < 0.001, ***P* < 0.01, or NS *P* > 0.05).

**
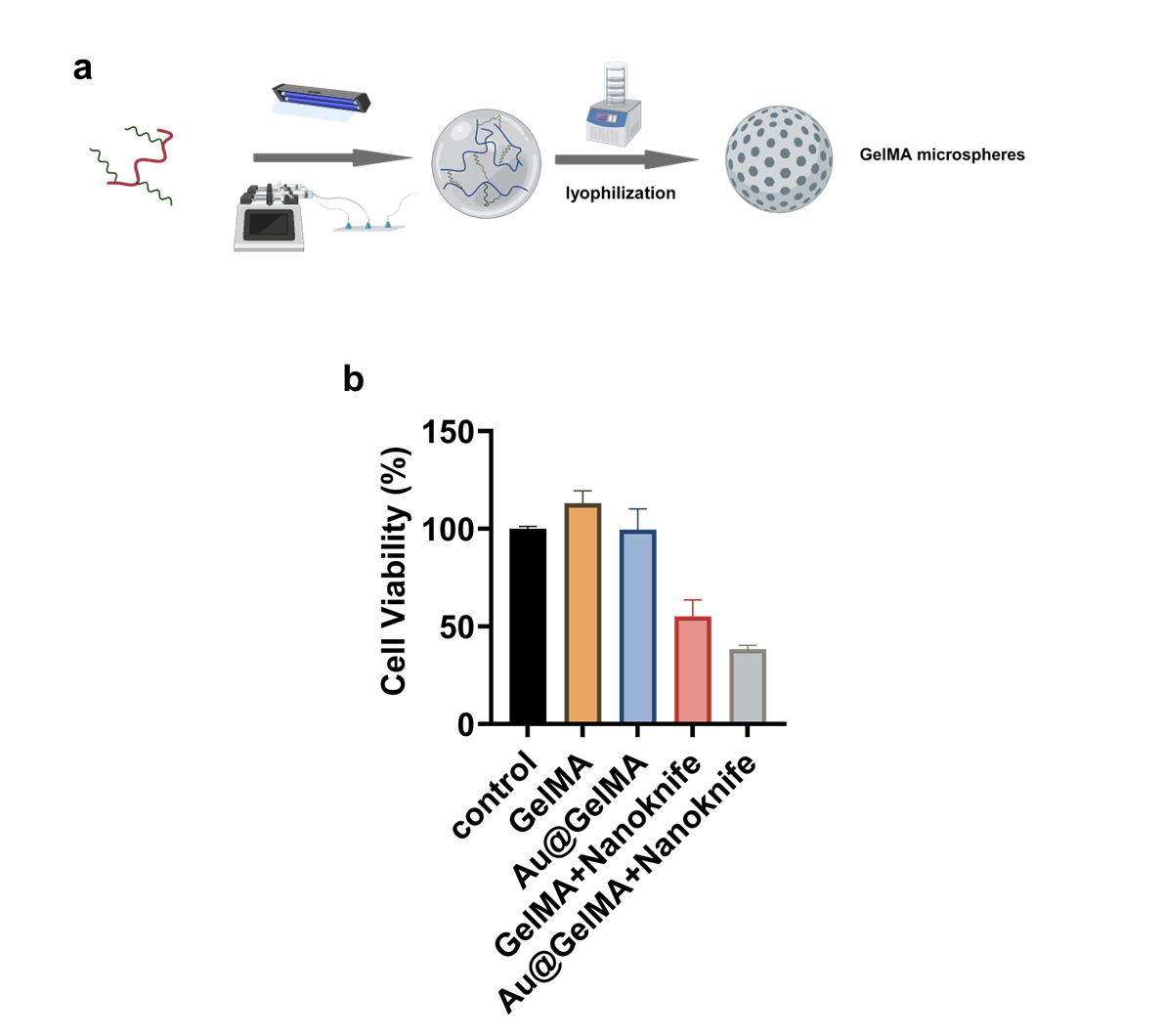
**

**Figure S5. a.** Scheme of the fabrication route of GelMA. GelMA microspheres were fabricated using microfluidics and followed by the UV light exposure for photo-crosslinking. Then, the porous GelMA microspheres were prepared after the lyophilization. **b.** *In vitro* anti-cancer efficiency of GelMA-based microspheres combined with Nanoknife (n = 2).

**
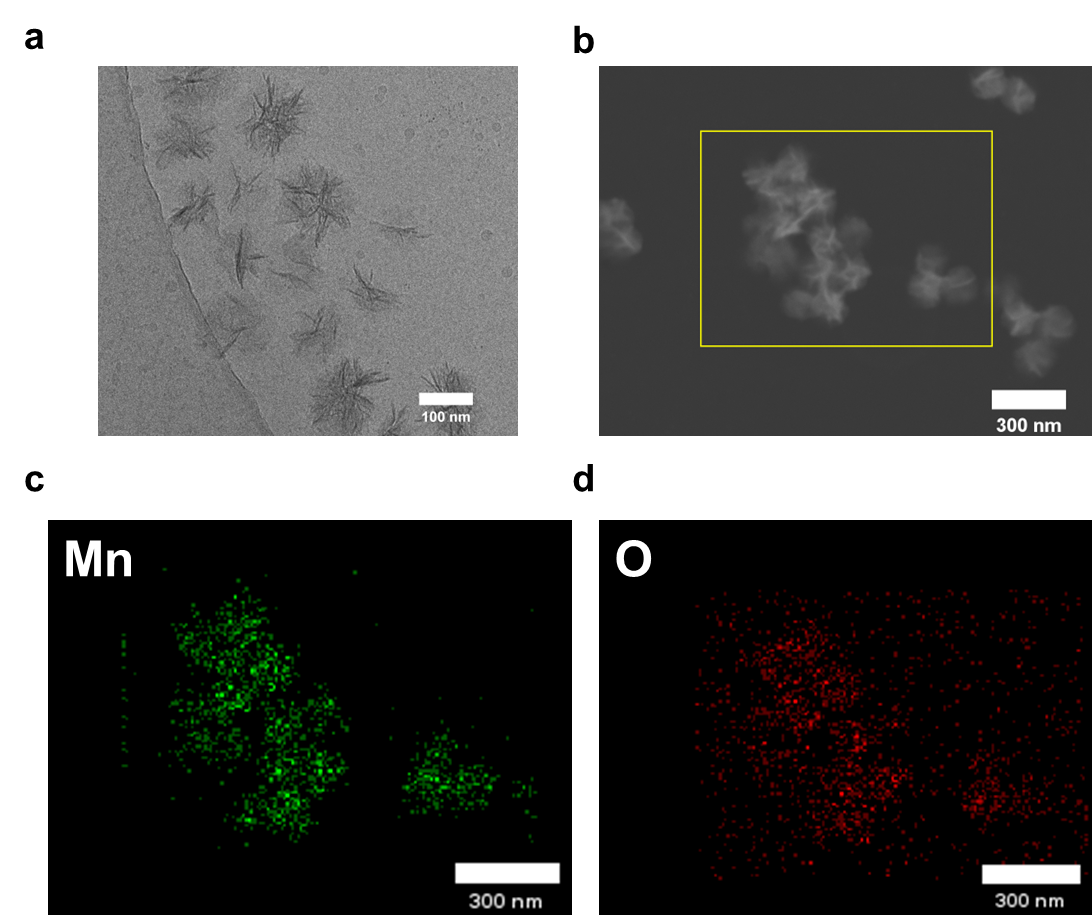
**

**Figure S6. a.** Representative cryo-TEM image of PM NPs. Scale bar: 100 nm. **b.** TEM image of PM NPs for EDX mapping. Scale bar: 300 nm. **c.** Element of Mn mapping on selected PM NPs. Scale bar: 300 nm. **c.** Element of O mapping on selected PM NPs. Scale bar: 300 nm.

**
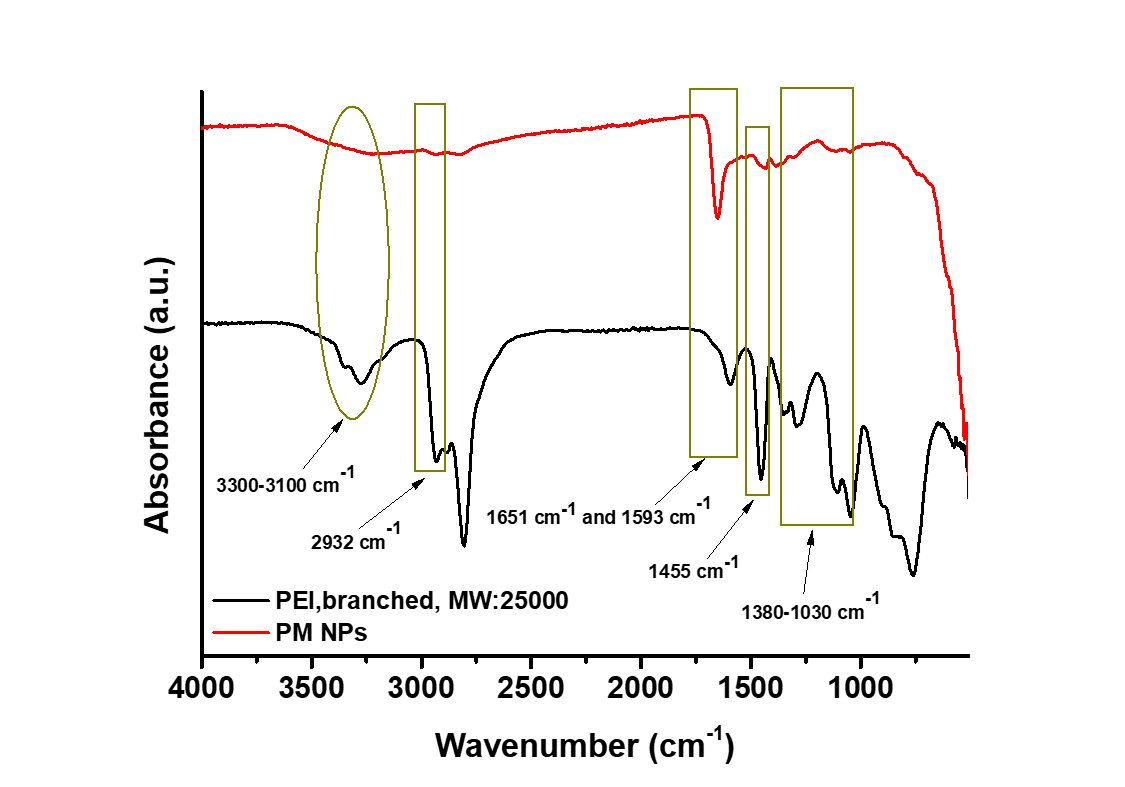
**

**Figure S7.** FTIR spectra of PEI and PM NPs.

**
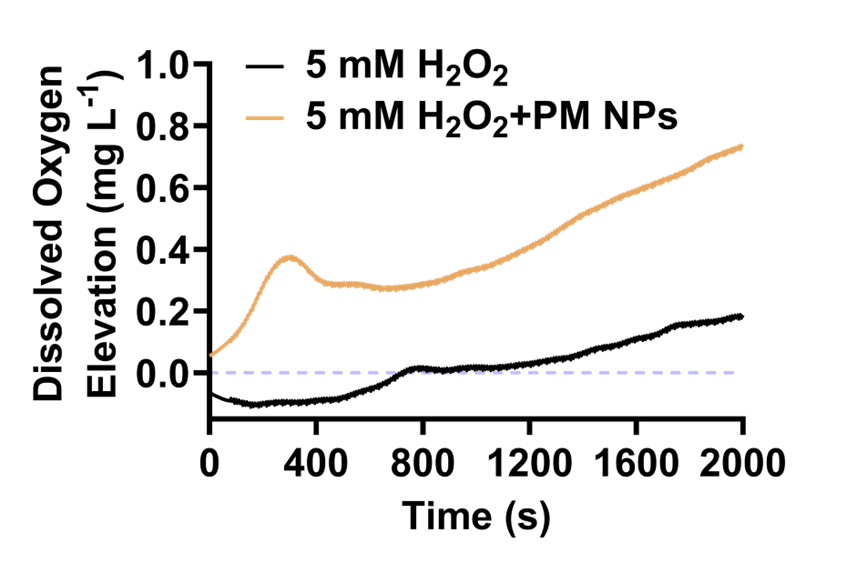
**

**Figure S8.** O_2_ generation profile of PM NPs incubated with H_2_O_2_ (5 mM).

**
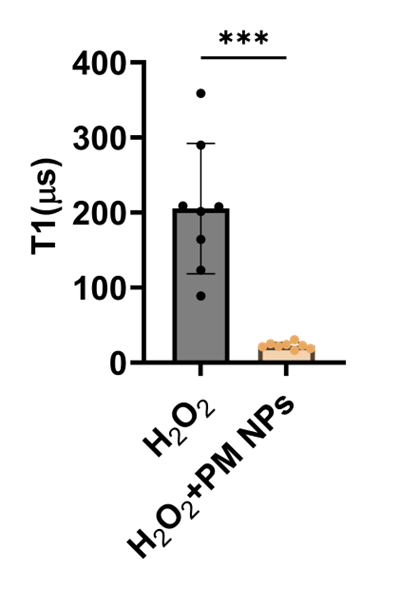
**

**Figure S9.** T1 relaxation time (μs) of FND particles in H_2_O_2_ solution (0.979 mM) with PM NPs (0.15 mg mL^-1^) or without. The results were extracted from the recorded data by biexponential fitting (n=8) [1]. Error bars are based on standard errors of the mean and statistical significance was determined by ANOVA. (****P* < 0.001, ***P* < 0.01, or NS *P* > 0.05).

**
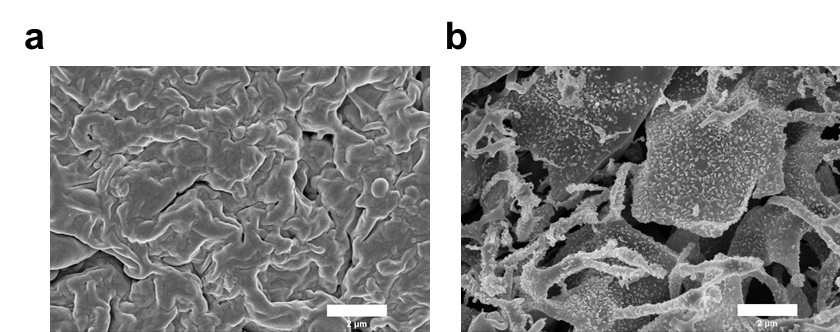
**

**Figure S10. a.** SEM image of Au@GelMA microspheres surface. Scale bar: 2 μm. **b.** SEM image of PM@Au@GelMA microspheres surface. Scale bar: 2 μm.

**
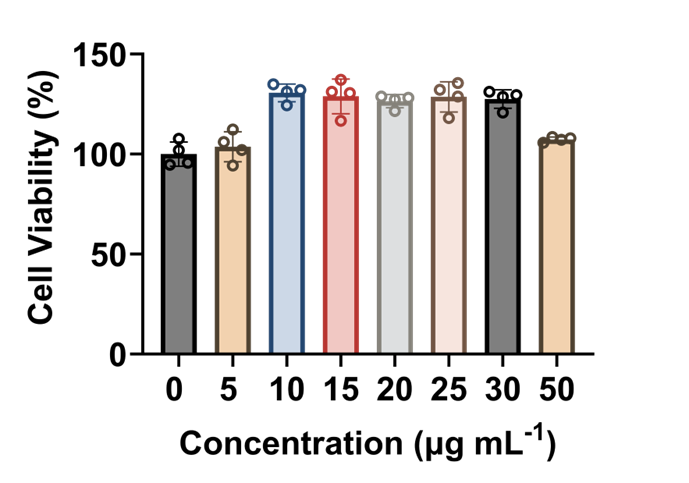
**

**Figure S11.** Cell viability of RAW cells after being incubated with PM NPs of different concentrations for 24 h.

**
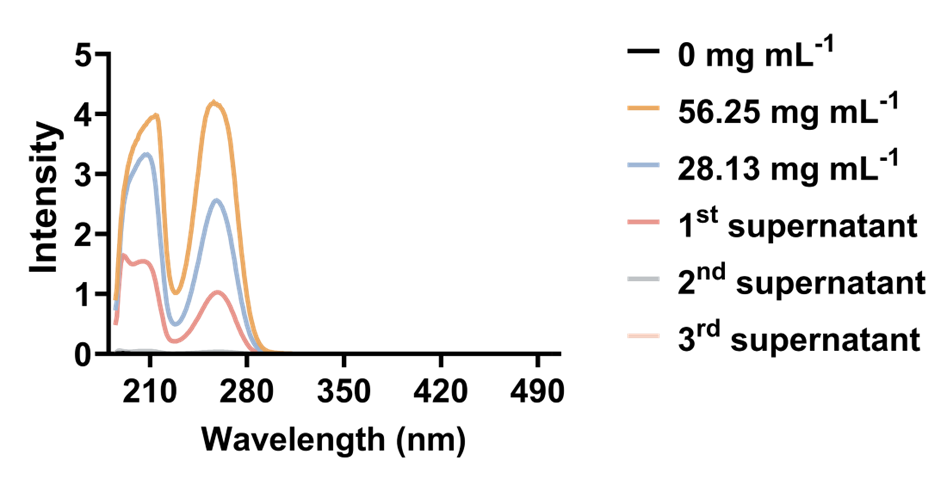
**

**Figure S12.** UV spectrums of c-di-AMP aqueous solution with different concentrations (0 mg mL^-1^, 28.13 mg mL^-1^, 56.25 mg mL^-1^) and those of the supernatants of c-d-AMP@PM NPs dispersion after three times centrifuges.

**
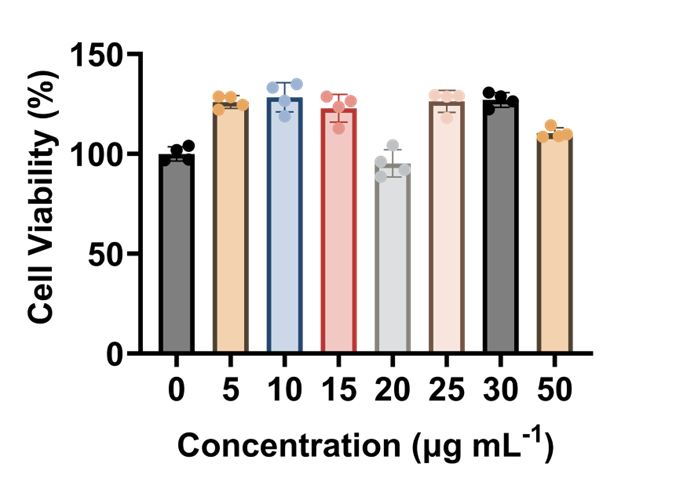
**

**Figure S13.** Cell viability of RAW cells after being incubated with c-di-AMP@PM NPs of different concentrations for 24 h.

**
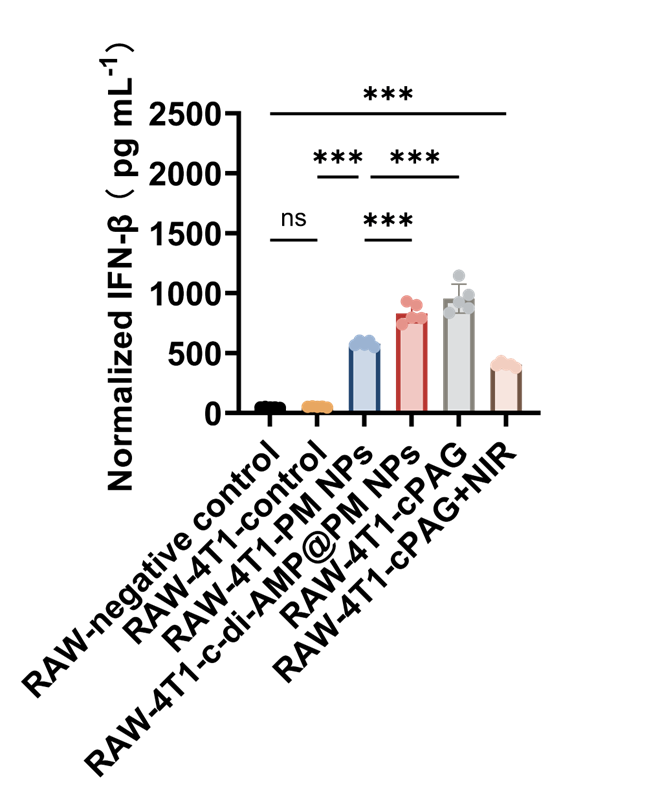
**

**Figure S14.** Normalized IFN-β secretion of RAW cells incubated with the supernatants that were collected from prolonged different treated 4T1 cells (n = 5).

**
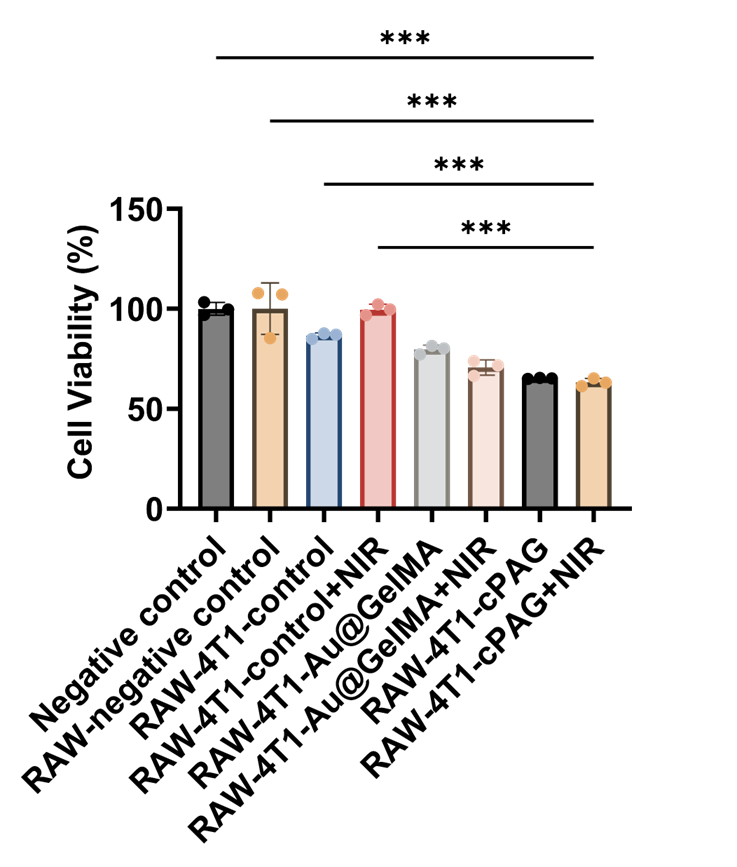
**

**Figure S15.** Cell viability of 4T1 cells after being cultured with supernatants collected from differently RAW cells, which had been stimulated by supernatant collected from prolonged different treated 4T1 cells (n = 3).

**
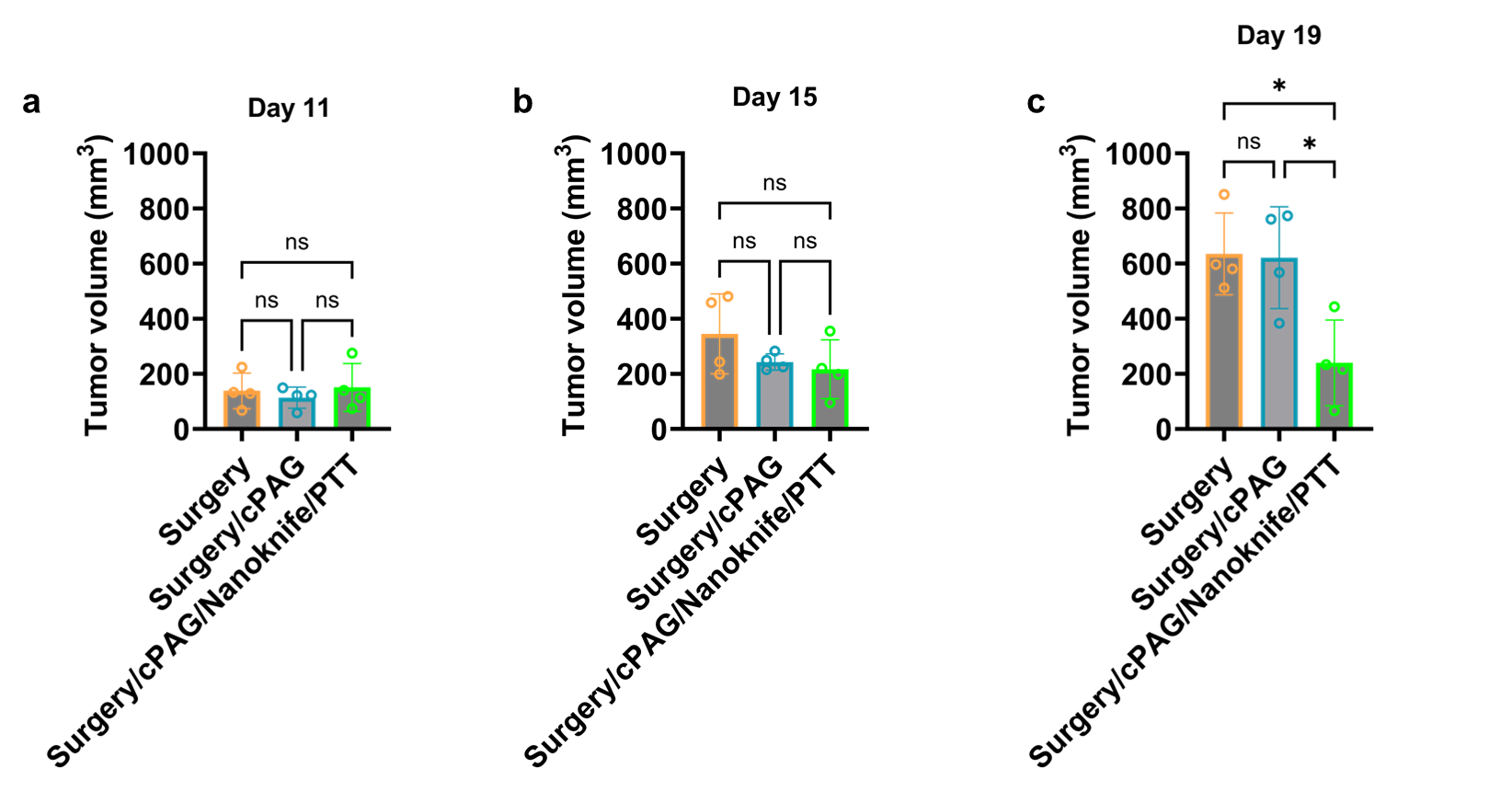
**

**Figure S16. a.** Statistics of tumor volumes in different groups (n = 4) on day 11. **b.** Statistics of tumor volumes in different groups (n = 4) on day 15. **c.** Statistics of tumor volumes in different groups (n = 4) on day 19.

**Reference**

[1] T.A. Vedelaar, T.H. Hamoh, F.P.P Martinez, M. Chipaux, and R. Schirhagl, , 2025. Optimizing data processing for nanodiamond based relaxometry, Advanced Quantum Technologies 8(4) (2025) 2300109.
